# Y-chromosomal analysis of Greek Cypriots reveals a primarily common pre-Ottoman paternal ancestry with Turkish Cypriots

**DOI:** 10.1371/journal.pone.0179474

**Published:** 2017-06-16

**Authors:** Alexandros Heraclides, Evy Bashiardes, Eva Fernández-Domínguez, Stefania Bertoncini, Marios Chimonas, Vasilis Christofi, Jonathan King, Bruce Budowle, Panayiotis Manoli, Marios A. Cariolou

**Affiliations:** 1 Department of Cardiovascular Genetics and The Laboratory of Forensic Genetics, The Cyprus Institute of Neurology and Genetics, Nicosia, Cyprus; 2 Department of Primary Care and Population Health, University of Nicosia Medical School, Nicosia, Cyprus; 3 Cyprus School of Molecular Medicine, The Cyprus Institute of Neurology and Genetics, Nicosia, Cyprus; 4 Department of Archaeology, Durham University, Durham, United Kingdom; 5 Department of Biology, University of Pisa, Pisa, Italy; 6 Center for Human Identification, University of North Texas Health Science Center, Fort Worth, Texas, United States of America; 7 Center of Excellence in Genomic Medicine Research, King Abdulaziz University, Jeddah, Saudi Arabia; Harvard Medical School, UNITED STATES

## Abstract

Genetics can provide invaluable information on the ancestry of the current inhabitants of Cyprus. A Y-chromosome analysis was performed to (i) determine paternal ancestry among the Greek Cypriot (GCy) community in the context of the Central and Eastern Mediterranean and the Near East; and (ii) identify genetic similarities and differences between Greek Cypriots (GCy) and Turkish Cypriots (TCy). Our haplotype-based analysis has revealed that GCy and TCy patrilineages derive primarily from a single gene pool and show very close genetic affinity (low genetic differentiation) to Calabrian Italian and Lebanese patrilineages. In terms of more recent (past millennium) ancestry, as indicated by Y-haplotype sharing, GCy and TCy share much more haplotypes between them than with any surrounding population (7–8% of total haplotypes shared), while TCy also share around 3% of haplotypes with mainland Turks, and to a lesser extent with North Africans. In terms of Y-haplogroup frequencies, again GCy and TCy show very similar distributions, with the predominant haplogroups in both being J2a-M410, E-M78, and G2-P287. Overall, GCy also have a similar Y-haplogroup distribution to non-Turkic Anatolian and Southwest Caucasian populations, as well as Cretan Greeks. TCy show a slight shift towards Turkish populations, due to the presence of Eastern Eurasian (some of which of possible Ottoman origin) Y-haplogroups. Overall, the Y-chromosome analysis performed, using both Y-STR haplotype and binary Y-haplogroup data puts Cypriot in the middle of a genetic continuum stretching from the Levant to Southeast Europe and reveals that despite some differences in haplotype sharing and haplogroup structure, Greek Cypriots and Turkish Cypriots share primarily a common pre-Ottoman paternal ancestry.

## Introduction

Cyprus, an eastern Mediterranean island, is located south of Turkey, west of Syria, north of Africa and east of Greece. The island’s prehistory dates as far back as the 11^th^ millennium BC, and recent archeological evidence claims the discovery of probably the oldest Mediterranean farming village in Southwest Cyprus[1]. Ancient Greeks (primarily Achaeans) started settling Cyprus during the Late Bronze Age[2]. The Phoenicians were known to have lived alongside the Greeks who with time had become Hellenized[3]. Cyprus’ privileged position, situated at the crossroads of three continents, resulted in a turbulent history dominated by many great empires. These powers included the Assyrians, Persians, Alexander the Great and his successors of the Ptolemy dynasty of Egypt and the Romans, all before Cyprus became part of the Byzantine Empire[4]. The conquest of the island continued later on through the crusaders of Richard the Lionhearted of England, the rule of the Frankish Lusignan family followed by the Venetian rule, three centuries of Ottoman rule (1571–1878), and finally the British rule until 1960, when Cyprus became an independent country[4].

The total population of Cyprus on the eve of the Ottoman takeover (1571) was around 200,000[5]. By the turn of the 17^th^ century a substantial Muslim minority had appeared in Cyprus with the total taxable population (only adult males) amounting to 20,000 Muslims and 85,000 Christians[6,7]. The Ottoman settlers of Cyprus comprised of both civilians (mainly craftsmen and other skilled workers) and soldiers and mercenaries of the Ottoman army [8]. These individuals were most likely a mix of indigenous Anatolian populations (possibly including some Armenians and Greeks) and Turkic populations of Central Asian origin who were already admixed with the local Anatolian population after their arrival in Anatolia during the 13^th^ cent. AD[9]

Previous population genetic studies have identified that both Greek Cypriots (GCy)[10–13] and Turkish Cypriots (TCy)[14] show genetic affinity with surrounding Southeast European and particularly Near Eastern populations. Despite historical records on the origins of GCy and TCy, the genetic ancestry of the two communities has not as yet been systematically compared. Generally, two different, but not mutually exclusive scenarios might hold. Scenario 1: TCy and GCy derive primarily from the same local paternal gene pool, diverging only recently (Ottoman era) as a result of Islamization and gradual formation of a separate TCy community. Scenario 2: TCy derive primarily from the mainland Turkish paternal gene pool, migrating to Cyprus during the Ottoman era of the island.

Genetics can play a significant role in describing the ancestry of the current inhabitants of Cyprus. A Y-chromosome analysis was performed to (i) determine paternal ancestry among the GCy community in the context of Southeast Europe and the Near East; and (ii) identify genetic similarities and differences between GCy and TCy in terms of paternal ancestry.

## Materials and methods

### Sample collection and DNA extraction

For the current study, 344 unrelated (no biological relationship) GCy were selected from the general population. The selection was performed stratified by district and included male GCy from all the seven main towns (both urban and rural regions) of the island: Nicosia (central) n = 78; Limassol (South) n = 75; Famagusta (East) n = 42; Larnaca (South East) n = 42; Paphos (South West) n = 27; Kyrenia (North) n = 42; and Morfou (North West) n = 38. Only individuals whose paternal grandfather had confirmed GCy ancestry were included. The project was approved by the Cyprus National Bioethics Committee. All participants gave their written consent. Automated DNA extractions were performed on a QIAGEN Universal Biorobot, using the QIAamp 96 DNA Swab BioRobot Kit following the manufacturer’s instructions.

### Y-chromosome genotyping

Y-short tandem repeat (STR) typing was performed using the PowerPlex^®^ Y23 System (Promega). This kit allows for simultaneous analysis of 23 loci on the Y-chromosome.

Automated PCR setup of the extracted DNA was performed on the QIAGEN Universal Biorobot. PowerPlex^®^ Y23 amplification was performed according to the manufacturer’s recommendations. PCR products were separated and detected on an ABI PRISM® 3130xl Genetic Analyzer, using POP-4^®^ Polymer. The results were analyzed with GeneMapper^®^ ID v3.2 software. Allele designation was in accordance with the bins and allelic ladder panels provided within the kit macro. These data have been submitted to the Y-STR Haplotype Reference Database (YHRD) and are now available under the following accession number:YA004186, as well as in S1 Table.

### Y-haplogroup determination and SNP genotyping

All 23-STR GCy haplotypes were input to the online Whit Athey’s Haplogroup Predictor tool[15] which generates probabilities for membership to one of the major Y-DNA haplogroups. In the current analysis, the ‘27-Haplogroup’ version of the Predictor was used and ‘Mediterranean’ was initially chosen under ‘area selection’, while the prediction was additionally repeated selecting all other areas (NW Europe, East Europe, South Asia, and ‘Equal Priors’) in turn. This was done as we wanted to avoid the assumption that none of the haplotypes in the dataset have a recent origin outside Cyprus. Another predictor tool[16], was also used for confirmation purposes. These two haplogroup prediction tools have been found to have a validity of 99% and 97%, respectively, in assigning major haplogroups and subclades[17]. Haplotypes that were assigned a haplogroup with a probability <90%, underwent specific Y-SNP genotyping to confirm the haplogroup. SNP genotyping was additionally used to determine specific subclades within major haplogroups.

iPLEX assay design and SNP genotyping assay design were based on published sequences retrieved from the National Center of Biotechnology Information (NCBI) databases. All selected sequences were submitted to MASSARRAY Assay Design 4.0 software (Sequenom Inc., San Diego, CA, USA). A 20-plex multiplex assay was developed, and the assay variants, primers and sequences are listed in S2 Table. For quality control purposes, all samples were genotyped in duplicate on separate plates and on separate days. Where the genotype call for each sample for the two separate amplifications was identical, the result was recorded. Where discrepancies were obtained, no genotype was assigned for the particular sample.

For comparison purposes, a publicly available Turkish Cypriot (TCy) population sample (n = 380 Y-STR haplotypes)[14] (YHRD accession number: YA003850) was analyzed (S3 Table). Although these TCy haplotypes were assigned haplogroups *in silico* in the original study using one of the tools used in the current study (Whit Athey’s Predictor), this procedure was repeated, following the same methodology used in the GCy population sample (described in detail above), with the only difference being that no SNP testing was performed, as the actual samples were not available. Additionally, the TCy haplotypes were compared with our SNP-confirmed GCy haplotypes, and the same haplogroup was assigned to any TCy haplotype that had an exact match (17/17 markers) or showed very high resemblance with SNP-confirmed haplotypes in the GCy sample. If a TCy haplotype remained unassigned even after comparison with SNP-confirmed GCy haplotypes, it was assigned to the most probable macro-haplogroup (e.g. F-unclassified, K-unclassified, etc.). The haplogroup assigning procedure in the TCy sample was initially performed ‘blinded’ to the original study’s haplogroup assignments, in order to avoid ascertainment (assessor) bias in haplogroup assignment. Following the haplogroup assignment in the TCy sample, the results were compared to those from the original study[14] and a very high degree of agreement (S3 Table) was observed, with minor differences being due to the slightly different assigning procedures followed. The Y-STR haplotypes and assigned Y-haplogroups (as well as details for the assigning procedure) for GCy and TCy population samples can be found in S1 and S3 Tables, respectively,

### Haplotype (STR) based analysis

A comparative database for ancestry-related STR-based analysis was built using publically available Y-STR profiles from populations in West Eurasia and North Africa. This database includes the aforementioned TCy sample[14], as well as a previously published sample of Greek Cypriots [12]. The TCy haplotypes were at a lower resolution than the GCy haplotypes (17- vs 23-STRs), so the database was restricted to the following 17 markers included in the AmpFlSTR® Yfiler® kit: DYS19, DYS385a/b, DYS389I/II, DYS390, DYS391, DYS392, DYS393, DYS437, DYS438, DYS439, DYS448, DYS456, DYS458, DYS635, and Y-GATA-H4. Database details can be found in S4 Table. For all population samples included in the dataset we followed the YHRD’s ethnic classification (https://yhrd.org/pages/resources/composition).

The number and percentage of individuals carrying GCy and TCy haplotypes in the populations from the 17-marker Y-STR haplotype database were calculated using Arlequin v3.5[18]. In order to increase the geographical resolution of the analysis, a manual search to detect additional shared GCy and TCy haplotypes in neighboring populations using the online YHRD tool was conducted (http://yhrd.org—Release 49, 2015/02/17). With the purpose of placing our shared haplotype analysis in historical context, the Time of Most Recent Common Ancestor (TMRCA) for a pair of shared AmpF*l*STR^®^ Yfiler^®^ Y-17 STR haplotypes was estimated with the online Clan McDonald TMRCA Probability Calculator using a mutation rate across all 17 Yfiler® STRs of 0.0025[19].

Rst genetic distances were calculated between GCy, TCy and different West Eurasian and North African populations (S4 Table) using both the YHRD online AMOVA tool and the software Arlequin v3.5. For the estimation of the square distance Rst using haplotypes with intermediate, duplicated and triplicated alleles were removed automatically by YHRD[20]. For the Arlequin analysis, the DYS458 locus was removed as it contained a large number of intermediate alleles, particularly prevalent among Cypriot haplotypes belonging to haplogroup J1 and this resulted in a non-random exclusion of a substantial proportion of our sample, potentially biasing the findings, especially regarding comparisons with populations rich in haplogroup J1. After excluding this marker, duplicated, triplicated and intermediate alleles were removed manually. This removal affected 27 (out of 344) haplotypes from the GCy population sample and 10 (out of 380) from the TCy population sample. The statistical significance of the Arlequin calculated Rst values was assessed using 10,000 permutations. In order to reduce random error and systematic error (selection bias), leading in turn to Type I and Type II errors, populations with very low sample size (n<30) were removed from the analysis. Pairwise Rst distances calculated between Cypriots and the other populations in the databases (S12 Table) were plotted as contour maps using Surfer version 9 (Golden Software, LLC). Only populations with a clear geographic location were included.

For comparison purposes, all analyses were repeated combining our newly typed GCy sample (n = 344), with the GCy sample from a previously published study[12] (n = 574 Y-STRs). Wherever applicable, results for the comparison between GCy and surrounding populations are presented in supplementary tables separately for our sample, the previously published GCy sample[12], and the combined GCy sample. In main tables and figures only results from our newly typed sample are presented, due to: (i) the lack of clarification regarding confirmation of Cypriot ancestry (i.e. two generations confirmed ancestry, as in the current study sample and the TCy sample)[14] in the previously published GCy sample (recruited primarily from a blood donation biobank); and (ii) potential bias due to decreased genetic diversity resulting from resampling from the same relatively small source possible (i.e. GCy). Regarding the second point, it should be noted that in population genetic studies, known relative are excluded from the study sample, something that was impossible to do in the specific example, since the two GCy samples were recruited independently. As a consequence, genetic diversity is reduced and the number of shared haplotypes is biased upwards, something that affects analyses such as AMOVA and shared haplotype analysis.

In order to reveal haplotype microvariations between GCy and TCy and quantify mutation steps and expansion times, a Y-STR based phylogenetic network of the most prevalent Cypriot haplogroups (J2a, J1, E-M78, E-M123, G2a, R1b) defined by 17 STR loci was constructed using the program Network 5.0.0.1 (Fluxus-Engineering) with the median joining algorithm. The 17 loci used were DYS19, DYS389 I, DYS389 II, DYS390, DYS391, DYS392, DYS393, DYS385a/b, DYS437, DYS438, DYS439, DYS448, DYS456, DYS458, DYS635 (Y-GATA-C4), and Y-GATA-H4. For the multi-copy short tandem repeat or microsatellite (STR) DYS389I,II, the DYS389b value was DYS389I subtracted from DYS389II. We have added labels to haplotypes having exact (17/17 STR) matches in surrounding populations. We have repeated this analysis after removing loci with high variability (weight <10, DYS458, DYS385a/b) and low variability (weight >90, DYS392, DYS438) in all haplogroups in order to minimize the number of reticulations. The following STRs were used in the 12-loci analysis: DYS389I, DYS448, DYS389b, DYS19, DYS391, DYS437, DYS635, DYS390, DYS439, DYS393, DYS456, YGATAH4.

The Y-STR loci were weighted based on the inverse of their variances and estimated times of expansion were calculated (wherever applicable) with two different mutation rates, based on Tofanelli et al (2016)[21] (mutation rate: 0,00309 x locus x generation) and repeated using an alternative formula by Burgarella et al (2011)[22] (mutation rate: 0,002668 x locus x generation). The MJN analysis was repeated using the combined GCy sample (n = 918), and presented in Supplementary material, as in all other STR-based analyses.

### Haplogroup based analysis

As with the Y-STR analyses, Y-haplogroup-based analyses were repeated by combining our newly typed sample (n = 344) with the aforementioned previously published GCy sample (n = 631 with SNP-confirmed Y-haplogroup). Therefore, Y-haplogroup frequencies were calculated separately for our GCy sample, the previously published GCy sample (10) and the combined GCy sample (total n = 975 with Y-haplogroup data) and are presented in supplementary tables and figures. For the reasons described in the preceding sub-section, results presented in main tables and figures are based on our newly typed sample only.

All studies reporting detailed Y-haplogroup data from populations surrounding Cyprus were considered[13,14,21,23–36] (S5 Table, S1 Fig). Our approach for determining haplogroup distributions comprised the following steps: (i) extracting the raw haplogroup data (absolute numbers) from each study; (ii) standardizing the nomenclature for each haplogroup [International Society of Genetic Genealogy (2016)]; (iii) merging data from different studies and summing the absolute frequency of each haplogroup for each population/sub-population; and (iv) calculating relative frequencies (%) of each haplogroup for each population/sub-population, using the merged data. Due to the fact that a key study containing Turkish and Kurdish samples from Central Anatolia[29] did not provide specific subclades for haplogroup E1b1b, a validated haplogroup prediction tool was used for determining those[16,17]. Y-haplogroup frequencies for Cypriots and for all other relevant populations can be found in S6 Table and S7 Table, respectively.

Fisher’s Exact test was used to detect the presence of statistically significant differences in haplogroup frequencies between these populations, using STATA v12 (StataCorp. 2011. Stata Statistical Software: Release 12. College Station, TX: StataCorp LP).

Principal Component Analysis (PCA) was performed on haplogroup frequencies using XLSTAT (Addinsoft, New York, NY 10001), and the first two principal components were plotted in a PCA plot. The PCA was repeated after simulating the pre-Ottoman Y-haplogroup composition of the Cypriot population. Since, based on previous publications[12,14], a small proportion of Cypriots appear to carry Eastern Eurasian haplotypes, the pre-Ottoman haplogroup composition of Cypriots was estimated, under the assumption of an Ottoman origin of some of the Eastern Eurasian haplogroups found in Cyprus (see Introduction for justification). The reason for focusing on the Eastern Eurasian haplogroups in our analysis is that Western Eurasian haplogroups, such as those mentioned above, were already present in Cyprus prior to the arrival of the Ottomans. Therefore only the presence of Eastern Eurasian haplogroups can clearly indicate, in both qualitative and quantitative terms, the influx of Ottoman Turks in the island. From all Eastern Eurasian Y-haplogroups detected (C-M130, H-L901, N-M231, O-M175 and Q-M242, R-M479), we have identified possible proto-Turkic haplogroups, based on: (i) a clear Central Asian (and more specifically Altaian) origin of the specific haplogroup[37,38] and (ii) presence of this haplogroup in modern day Turks[28]. The identified possible proto-Turkic haplogroups fulfilling both criteria were C-M130, N-M231, and Q-M242). Predicted pre-Ottoman haplogroup frequencies were estimated by replacing these haplogroups (n = 16 for TCy; n = 2 for GCy) with other Y-haplogroups found among Cypriots, in a weighted manner. More specifically, after zeroing the frequency of all possible proto-Turkic haplogroups, the relative frequency (%) of all other haplogroups was multiplied by the total number of possible proto-Turkic haplotypes for each population (GCy and TCy) separately, giving thus (after rounding up) a predicted number of haplotypes belonging to each haplogroup (shown in red in S8 Table). The procedure for assigning new haplogroups and the predicted pre-Ottoman Y-haplogroup composition of Cypriots can be found in S8 Table.

## Results

### Intra-population (Greek Cypriot) analysis

Three hundred and fourteen Y 23-STR haplotypes were unique amongst the 344 newly typed GCy samples (91%). From the remaining 30 haplotypes, 27 were observed in 2 individuals, 2 in 4 individuals and 1 in 3 individuals. Within this population, non-standard alleles were observed at the DYS19, DYS549, DYS643, DYS533, and DYS392 loci, where 12 samples presented duplications, and for one sample the DYS385 locus presented three alleles. Seventeen STR loci from our GCy sample were compared to the same 17 STR loci that were used to type a previously published GCy study population (n = 574). There were 87 matching haplotypes between the samples from these two GCy studies; in other words, 25% of the 17-STR haplotypes in the two independent GCy study populations were identical. Haplotype diversity values of our GCy sample, as well as the previously published TCy sample and other Greek and Turkish populations from our Y-17 comparative database can be seen in S9 Table. Noticeably, the GCy and TCy populations displayed generally low percentages of different haplotypes compared to other populations, while haplotype diversity values were all in the range of 0.99.

A total of 13 different major Y-haplogroups (E1b1b, G1, G2, J1, J2, I1, I2, L, N, Q, R1a, R1b, T) were found among the 344 newly typed GCy haplotypes. [12] The most frequent was haplogroup J2 (29.6%), followed by haplogroup E1b1b (26.8%). Other common haplogroups were G2 (12.5%), R1b (11.9%), and J1 (8.7%). The remaining haplogroups were found at frequencies <5% (S6 Table).

### Inter-population analysis

**Fig 1:**
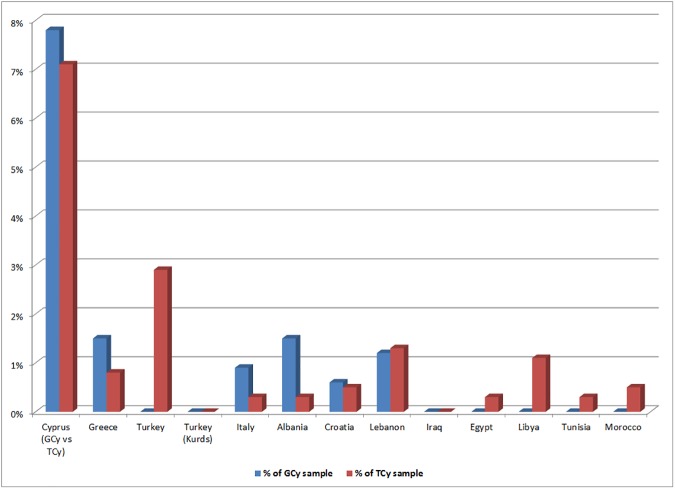
**Shared Greek Cypriot (a) and Turkish Cypriot (b) haplotypes in Southeast Europe, the Near East and North Africa.** The bar charts represent the frequency of individuals sharing haplotypes as a proportion (%) of the total haplotypes in populations surrounding Cyprus. Population codes as in S5 Table.

There were 24 different 17-STR shared haplotypes between the newly typed GCy population sample and the previously published TCy sample. These haplotypes were carried by 27 of 344 (7.8%) GCy and 27 of 380 (7.1%) TCy individuals. Greek Cypriots also share haplotypes with other population from Southeast Europe and the Near East, namely Greeks (carried by 1.5% of individuals in the GCy sample and 0.5% of individuals in the Greek sample), Albanians (1.5% of GCy individuals), Lebanon (~1% of GCy individuals and Lebanese) and Italy (~1% of GCy and 0.1% of Italians). TCy, in addition to the shared haplotypes with GCy (7.1% of TCy sample), also share haplotypes with mainland Turks (carried by ~3% individuals in the TCy sample, ~1% of individuals in the Turkish sample), Lebanese (~1.5% of TCy, 1% of Lebanese), Greeks (1% of TCy, 0.5% of Greeks), and Libyans (1% of TCy), (Table 1), It should be noted that Table 1 shows comparisons with populations from the regions of Southeast Europe, Near East, and North Africa only. Comparisons between Cypriots and a wider selection of populations from Western Eurasia can be found in S10 Table. Fig 1 displays the percentage of individuals in different populations carrying (a) GCy (blue) and (b) TCy (red) haplotypes. Considering a generation to represent 28 years, the estimated coalescence time for a haplotype pair sharing 17/17 Y-STRs was estimated to be 1008 years, with 95% confidence (see Methods section for details). S11 Table shows all GCy-TCy shared haplotypes, together with the assigned Y-haplogroups, as well as Cypriot haplotypes having 17/17 matches with surrounding populations.

**Table 1:** Number and percentage of individuals from Southeast Europe, the Near East and North Africa that share haplotypes^a^ with Cypriots.

Greek Cypriots (n = 344) *vs*	Total sample n	Shared haplotypes, n (%)	Turkish Cypriots (n = 380) *vs*	Total sample n	Shared haplotypes, n (%)
Cyprus [Turkish Cypriots]	380	27 (7.8%)	Cyprus [Greek Cypriots]	344	27 (7.1%)
Greece [Greek]	538	5 (1.5%)	Turkey [Turkish]	1350	11 (2.9%)
Albania [Albanians]	502	5 (1.5%)	Lebanon [Lebanese]	555	5 (1.3%)
Lebanon [Lebanese]	555	4 (1.2%)	Libya [Libyan]	413	5 (1.3%)
Italy [Italian]	3023	3 (0.9%)	Greece [Greek]	538	3 (0.8%)
Bulgaria [Bulgarian]	318	2 (0.6%)	Croatia [Croatian]	1339	2 (0.5%)
Croatia [Croatian]	1339	2 (0.6%)	Casablanca Morocco [Moroccan]	166	2 (0.5%)
South Italy (Arbëreshë, Italo-Albanians)	132	1 (0.3%)	Italy [Italian]	3023	1 (0.3%)
South-East Romania [Romanian]	122	1 (0.3%)	South Italy (Arbëreshë, Italo-Albanians)	132	1 (0.3%)
Turkey [Turkish]	1350	0 (0.0%)	Bulgaria [Bulgarian]	318	1 (0.3%)
Israel [Israeli Muslim]	119	0 (0.0%)	Israel [Israeli Muslim]	119	1 (0.3%)
Jordan [Arab]	164	0 (0.0%)	Egypt [Egyptian]	208	1 (0.3%)
Iraq [Iraqi]	124	0 (0.0%)	Tunisia [Tunisian]	218	1 (0.3%)
Tunisia [Tunisian]	218	0 (0.0%)	South-East Romania [Romanian]	122	0 (0.0%)
Libya [Libyan]	413	0 (0.0%)	Albania [Albanians]	502	0 (0.0%)
Egypt [Egyptian]	208	0 (0.0%)	Jordan [Arab]	164	0 (0.0%)
Casablanca Morocco [Moroccan]	166	0 (0.0%)	Iraq [Iraqi]	124	0 (0.0%)

^a.^Shared haplotype % represents the proportion of individuals among Greek Cypriots (n = 344) and Turkish Cypriots (n = 380) having an exact 17/17 Y-STR haplotype match in the specified populations (YHRD and comparative dataset).

Of the 24 GCy-TCy shared haplotypes, 20 are Cyprus-specific (i.e., not found in any other part of the world). In addition, 4/24 shared haplotypes belonging to Y-haplogroup J1 had the rare DYS458 19.2 microvariant while 2/24 GCy-TCy shared haplotypes, belonging to Y-haplogroup J2a, had the very rare allele 8 in loci DYS391 and YGATAH4. Finally, one GCy-TCy shared haplotype, belonging to Y-haplogroup G2a, had the rare duplication “15,16” at the DYS19 locus[39].

Calculated Rst genetic distances among pairs of populations can be found in Table 2 (also S12 Table; S2 Fig). Under a stepwise mutation model and when populations are highly sub-structured, Rst distance offers a reliable measure of population differentiation as it is independent of the mutation rate[40]. In this analysis, GCy and TCy show a very small genetic differentiation (Rst = 0.0008). Other than this observation, for both GCy and TCy, the lowest Rst values were with the Lebanese (Rst = 0.0080 and 0.0063, respectively) and Greeks (Rst = 0.0113, 0.0098, respectively). Comparisons between GCy and TCy produced non-significant p-values (>0.05) through permutation, indicating a single panmictic gene pool. It should be noted that a small sample of Calabrian Italians (Belvedere, n = 30) showed remarkably low, non-significant, genetic differentiation from both GCy and TCy (results not shown in Table 2 due to small sample size, but can be found in S12 Table).

**Table 2:** Pairwise genetic distance (Rst) between Cypriots and populations from Southeast Europe, the Near East and North Africa.

**Greek Cypriots *vs*^a^**	**n^b^**	**Rst^c^**	**Turkish Cypriots *vs*^a^**	**n^b^**	**Rst^c^**
Cyprus [Turkish Cypriots]	380	0.0008*	Cyprus [Greek Cypriots]	344	0.0008*
Lebanon [Lebanese]	505	0.0080	Lebanon [Lebanese]	505	0.0063
Greece [Greek]	405	0.0113	Greece [Greek]	405	0.0098
South-East Romania [Romanian]	122	0.0205	Iraq [Iraqi]	124	0.0168
Iraq [Iraqi]	124	0.0219	South-East Romania [Romanian]	122	0.0175
Iran [Iranian]	160	0.0269	Iran [Iranian]	160	0.021
Israel [Israeli Christian]	44	0.0296	Turkey [Turkish]	248	0.0229
Turkey [Turkish]	248	0.0316	Puglia Italy [Italian]	160	0.0271
Puglia Italy [Italian]	160	0.0319	Marche Italy [Italian]	138	0.0317
Marche Italy [Italian]	138	0.0359	Israel [Israeli Christian]	44	0.0354
**Additional YHRD sample comparisons**^d^					
**Greek Cypriots *vs***^a^	**n**^b^	**Rst**^c^	**Turkish Cypriots *vs***^a^	**n**^b^	**Rst**^c^
Cyprus [Turkish Cypriots]	380	0.0010*	Cyprus [Greek Cypriots]	344	0.0010*
Northern Greece, Greece [Greek]	191	0.0108	Reggio di Calabria, Italy [Italian]	74	0.0054*
Reggio di Calabria, Italy [Italian]	74	0.0175	Northern Greece	191	0.0081
Athens, Greece [Greek]	148	0.0191	East Anatolia [Turkish]	37	0.0130
East Anatolia [Turkish]	37	0.0232	Athens, Greece [Greek]	148	0.0147
Black Sea region [Turkish]	103	0.0271	Black Sea region [Turkish]	103	0.0161
Turkey [Turkish]	320	0.0336	Marmara Region, Turkey [Turkish]	385	0.0238
Marmara Region, Turkey [Turkish]	385	0.0339	Southeastern Anatolia, Turkey [Turkish]	150	0.0240
Southeastern Anatolia, Turkey [Turkish]	150	0.0349	Turkey [Turkish]	320	0.0242
Çukurova, Turkey [Turkish]	249	0.0646	Çukurova, Turkey [Turkish]	249	0.0487
*Greece [Greek] combined*^e^	595	0.0079	*Greece [Greek] combined*^e^	595	0.0061
*Turkey [Turkish] combined*^e^	1460	0.0338	*Turkey [Turkish] combined*^e^	1460	0.0246

a. Pairwise comparisons between Cypriots and other populations calculated by Arlequin, sorted from smallest to largest Rst for each (the smaller the Rst the lower the genetic differentiation)

b. Top 10 closest populations shown (only samples with n>30 shown)

c. Results from small samples (n<100) should be interpreted with caution

d. Includes additional Greek, Turkish and Calabrian population samples not included in the comparative dataset (sorted from smallest to largest Rst)

e. Combined includes all Y-filer (17 Y-STR) haplotypes from Greece and Turkey respectively, present in YHRD at the time of analysis

Asterisked(*) Rst values indicate non-significant differentiation (p-value ≥ 0.05), calculated using 10,000 permutations

Turks showed larger genetic differentiation from both GCy (Rst = 0.0316) and TCy (Rst = 0.0229). The P-values between Cypriots (both GCy and TCy) and Greeks and between Cypriots (both GCy and TCy) and Turks demonstrated statistically significant differences, indicating population structuring (i.e., evidence of two independent “fixed” populations).

Repeating this analysis comparing our GCy sample to a previously published GCy sample[12] for validation purposes, revealed that the pairwise genetic distance between these two random samples of GCy was very low (Rst = 0,0010) and statistically non-significant (S12 Table), confirming a single panmictic gene pool. When repeating the above analyses combining our GCy sample to the aforementioned previously published GCy sample, TCy still appeared the closest population to GCy, with the p-value being marginally significant. This could be attributed to the limitations relevant to combining our sample with the previously published GCy sample, described in the Methods section. Differences between the combined GCy sample and other populations were also in agreement to those observed in the original analysis (S12 Table).

When YHRD was used to estimate Rst values between GCy, TCy and specific Turkish and Greek sub-population samples not publicly available in the literature (and thus not included in our comparative dataset), the Turkish sub-populations showed higher genetic differentiation from Cypriots (GCy and TCy) than Greek sub-populations did (Table 2). Overall, the combined YHRD sample from Greece appeared much closer to Cypriots than the combined YHRD sample from Turkey. In addition, a different YHRD Calabrian sample (Reggio di Calabria) was included in order to confirm the very low genetic differentiation between Cypriots and Calabrians and indeed the Rst was very low particularly among TCy (Table 2). It should be noted that direct comparisons between Arlequin and YHRD results should be considered with caution due to methodological differences

**Fig 2:**
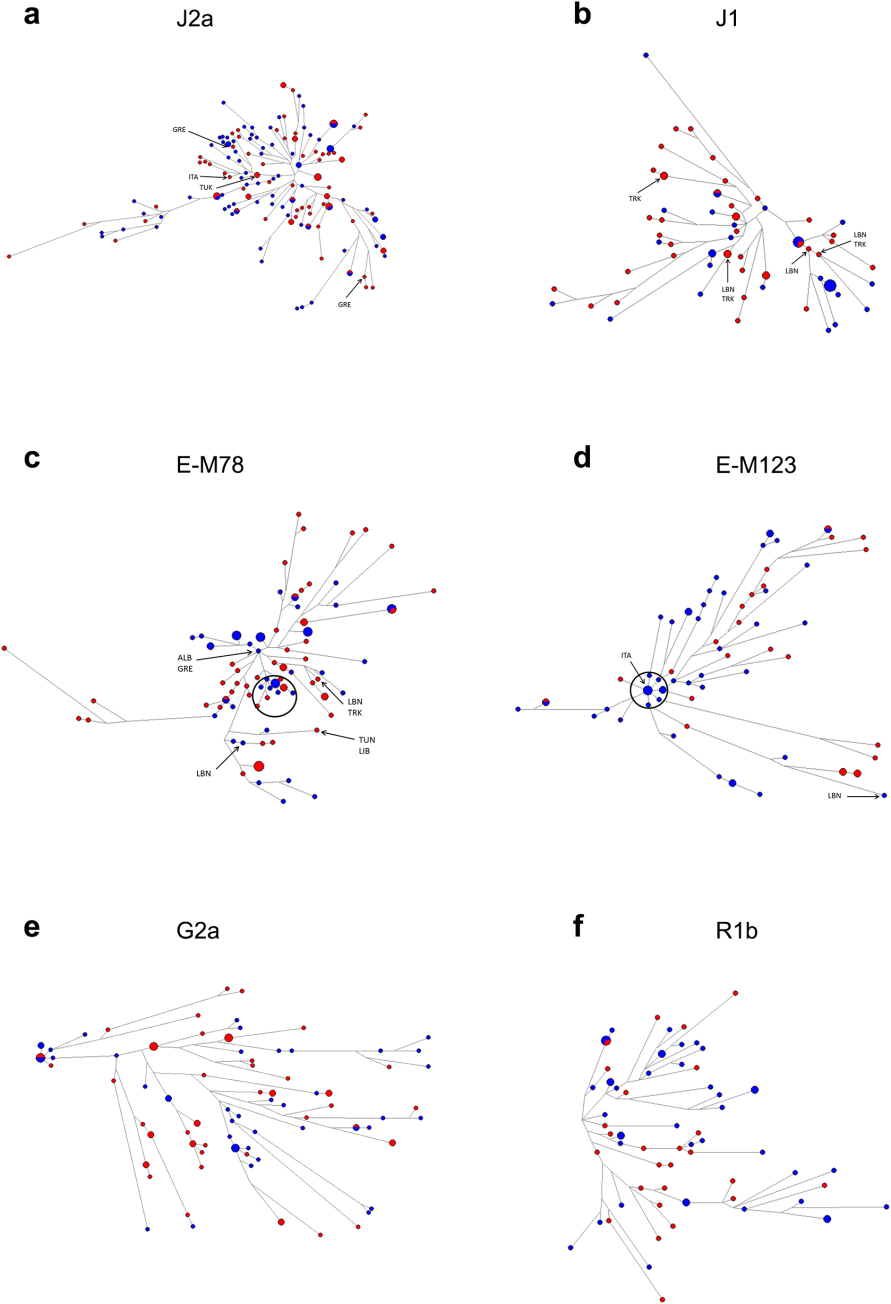
**Median-joining networks for haplogroups (a) J2a, (b) J1, (c) E-M78, (d) E-M123, (e) G2a, and (f) R1b.** Median Joining Network based on 17 Y-STR loci. For each network, blue colour indicates GCy haplotypes and red colour TCy haplotypes. Circles are sized according to the number of individuals sharing the haplotype, with the smallest circles representing one individual. The lengths of the connecting lines are proportional to the number of mutational steps separating two haplotypes. Haplotypes shared with surrounding populations are labelled with the corresponding country where the exact 17/17 haplotype match is found (GRE: Greece, TUR: Turkey, LEB: Lebanon, EGY: Egypt, LIB: Libya, TUN: Tunisia, ALB: Albania, CRO: Croatia)

The MJN analysis revealed that GCy haplotypes (coloured blue in Fig 2) appear in a more basal position than TCy haplotypes.(coloured red in Fig 2) in specific haplogroups, such as E-M78, while other haplogroups do not have any specific structure. Only two haplogroup networks (E-M123 and E-M78) reveal star-like agglutinations of some haplotypes or sub-clusters compatible with population expansion events (black ovals in Fig 2). Time expansions for these were estimated at 4018 years ago (+/- 507 DS) and 753 years ago (+/- 329 DS), respectively. Repeating the analysis using 12 STR loci, resulted in somewhat differently shaped networks, which however provided the same results, with only the aforementioned haplogroups showing evidence of star-like constructions (S3 Fig). Expansion times using an alternative formula (see Methods) and using 12-loci were very similar to those reported above. Overall, haplotype variation appeared to overlap between GCy and TCy, with no clear clustering of haplotypes based on ethnic background observed.

### Y-haplogroup analysis

**Fig 3:**
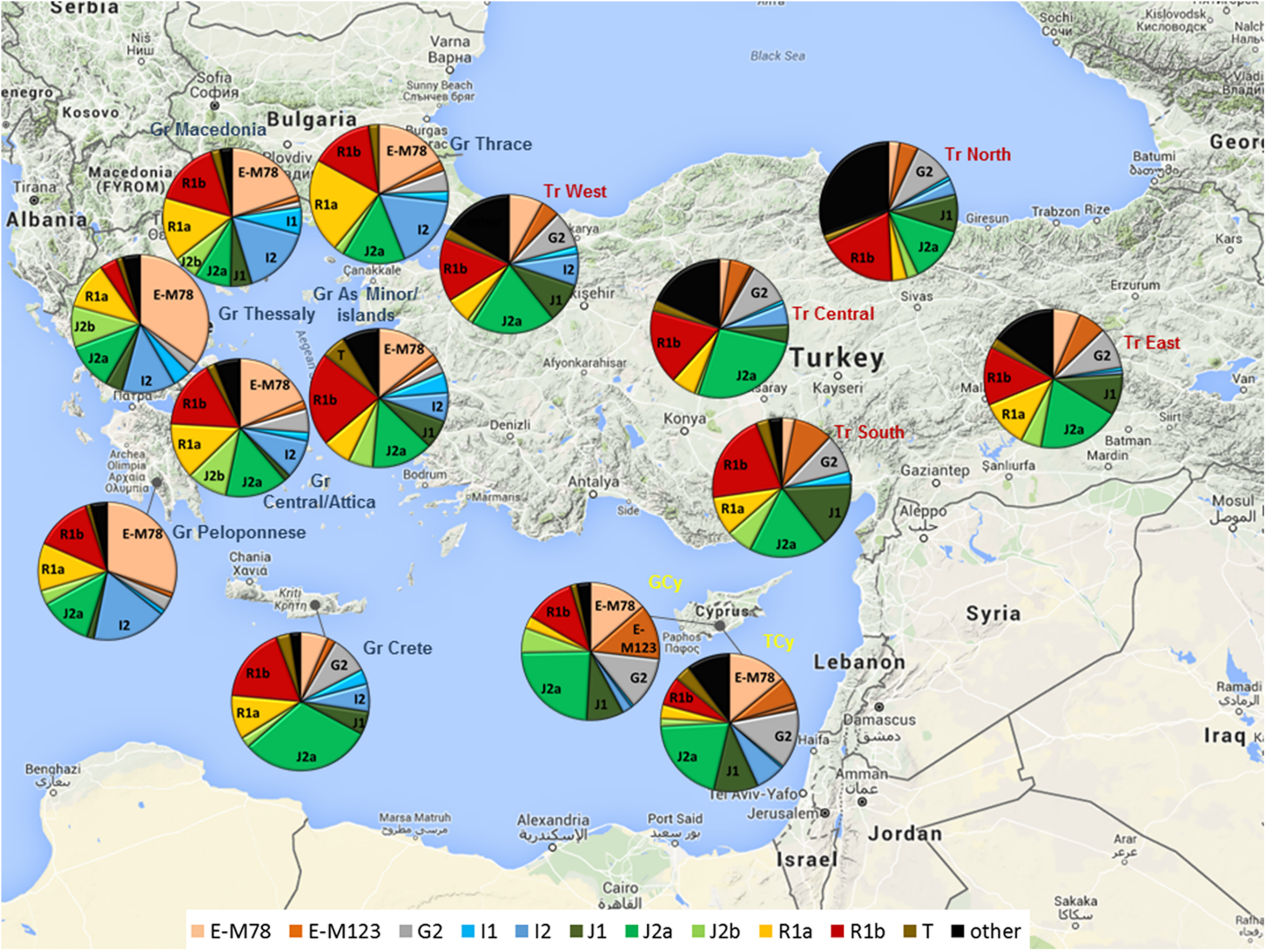
Y-haplogroup frequencies among Greek Cypriots, Turkish Cypriots, Greeks and Turks. Population codes as in S5 Table.

Y-haplogroup frequencies within GCy and TCy can be found in S6 Table. Y-haplogroup frequencies of Cypriots, Greeks, and Turks, as well as other surrounding populations can be found in Fig 1 (as well as S7 Table). GCy and TCy showed very similar frequencies for the major Y-haplogroups, differentiating both from Greek and Turkish sub-populations (Fig 3). The most frequent major Y-haplogroup subclade in both GCy and TCy was J2a-M410 (23.8% and 20.3% among GCy and TCy, respectively), followed by E-M78 (12.8% Vs 13.9%) and G2-P287 (12.5% Vs13.7%). R1b-M343 was found in higher frequency among GCy (11.9%) than TCy (6.8%), while the same applies for E-M123 (13.1% Vs 6.3%). Finally, haplogroup, although in much lower frequencies than the aforementioned haplogroups, haplogroup I2 was somewhat higher among TCy (6.8%), than among GCy (2.3%), while haplogroup J2b was higher among GCy (5.8%) than TCy (1.8%). Other, less common haplogroups (i.e. I1, R1a, L, and T) showed similar frequencies (in the range of 1–5%) between GCy and TCy.

One additional difference between GCy and TCy was the presence of moderate numbers of East Eurasian (primarily Central Asian) Y-haplogroups and small numbers of North African Y-haplogroups among TCy but not among GCy. The frequency of East Eurasian haplogroups among TCy was C-M130 (0.5%), H-L901 (0.3%), N-M231 (2.4%), O-M175 (0.8%) and Q-M242 (1.3%), reaching a total of 5.6%, but only totalling 0.6% among GCy. North African haplogroups (E-M81, E-V38) were only found among TCy (2.1%) (S6 and S7 Figs).

A major feature differentiating Cypriots from Greeks, is the much lower frequency of haplogroups I (2.9% GCy, 7.3% TCy, ~10–21% mainland Greeks) and R1a (2.9% GCy, 3.2% TCy, ~10–22% mainland Greeks) among the former. All differences in haplogroup frequencies between populations were statistically significant (Fisher’s Exact test, p<0.001).

**Fig 4:**
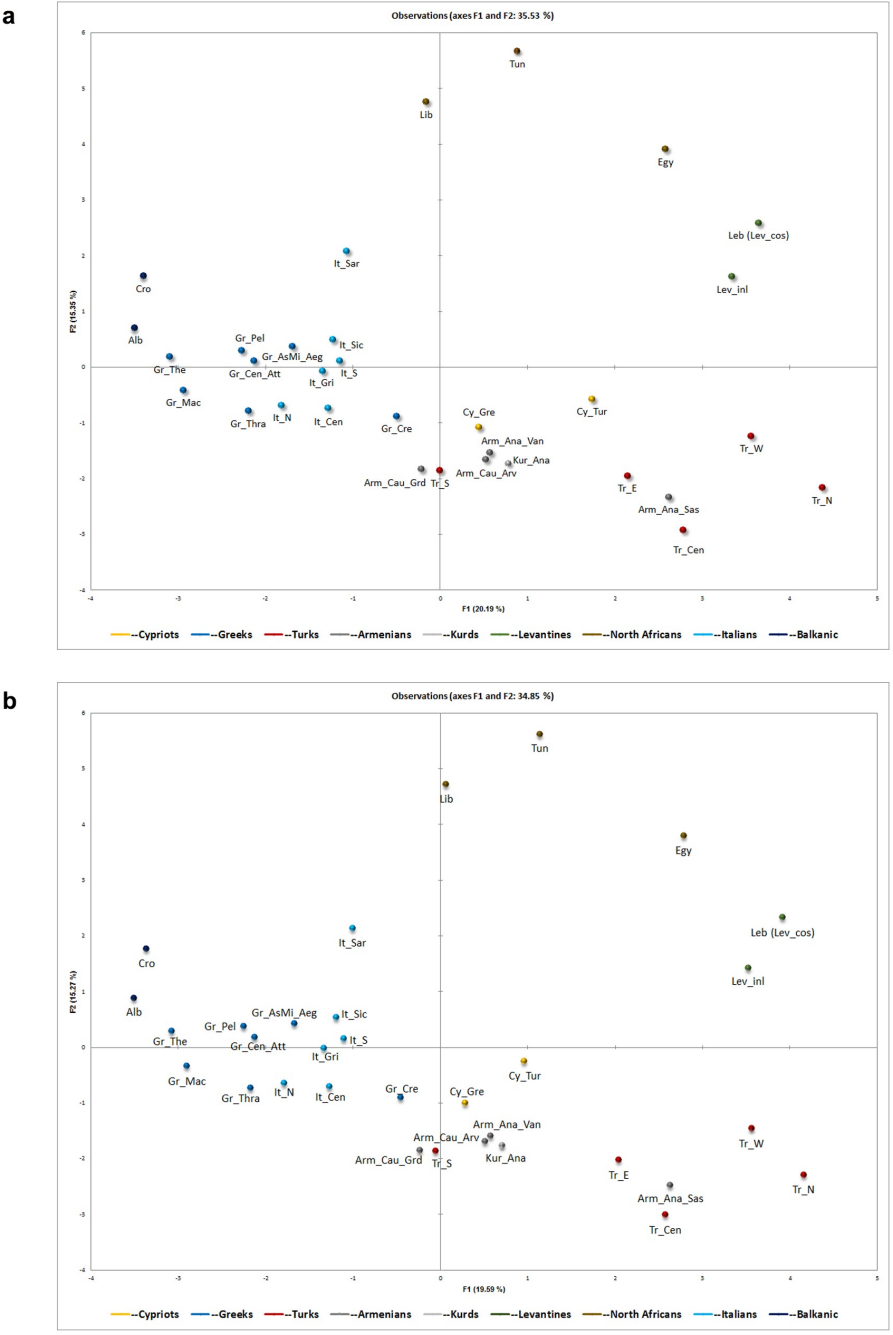
PCA plots based on Y-haplogroup frequencies showing relative genetic distances between Cypriots and surrounding populations. PCA plots based on Y-Haplogroup frequencies showing relative genetic distances between Cypriots, Greeks, Turks and other populations and sub-populations from the Central and Eastern Mediterranean and Near East regions (a), and after predicting the pre-Ottoman Y-haplogroup distribution of Cypriots, assuming no possible proto-Turkic admixture into Cyprus (b). Population codes as in S5 Table.

Principal Component Analysis (PCA) based on frequencies of 36 Y-haplogroups and subclades of 32 populations and sub-populations (Fig 4A) revealed that in terms of current patrilineage distribution, GCy cluster with some Armenian and Anatolian Kurdish groups and to a lesser extent South Turks and Cretan Greeks, while TCy, do not seem to cluster with other populations in terms of current haplogroup composition. When the same analysis was repeated by excluding possible proto-Turkic haplogroups among GCy and TCy (i.e. simulating the pre-Ottoman Y-haplogroup composition of the island), GCy and TCy were much closer in the PCA plot, with the TCy closely associated with the aforementioned cluster of GCy, Armenians, Kurds, South Turks and Cretan Greeks (Fig 4B).

## Discussion

### Shared paternal ancestry among Cypriots (GCy compared to TCy)

GCy and TCy share between them many more Y-chromosome haplotypes, both in relative and absolute terms, than with any other surrounding population. Given that historical and archaeological evidence shows the GCy to have been established on the island at the beginning of the Iron Age (ca. 1000 B.C.)[2] and the TCy at the beginning of the Ottoman era (ca. 1600 A.D), this high percentage of shared haplotypes between them could be explained either by a common local (pre-Ottoman) ancestry for both communities and a recent (few centuries) divergence (scenario 1 in Introduction), or a non-local (i.e. Turkish) paternal origin of TCy and extensive mixing with the local GCy population during the Ottoman era (scenario 2 in Introduction).

As indicated in Table 1, 7–8% of GCy and TCy share haplotypes between them. This is considerably larger than the sharing observed with other populations, but lower than the 25% haplotype sharing between our GCy sample and a previously published GCy sample[12]. This does not necessarily indicate substantial genetic differentiation between the two communities, due to the effect of resampling, described in the Methods section, Briefly, when a population sample is created by combining two separately recruited random samples from the same source population of relatively small size (e.g. GCy) there will be less genetic diversity and more haplotype sharing than in a single sample of the same size, since in any sample recruited for population genetics purposes, known relative are (or should be) excluded. As a proof of this phenomenon, the combined GCy sample has a lower percentage of different haplotypes (75%) than our newly typed sample (85%), which is paradoxical, since larger samples are normally expected to have higher % of different haplotypes. Therefore the 25% haplotype sharing between two independent GCy samples, is very likely biased upwards, thus cannot be used as a reference point for the extent of sharing between GCy and TCy.

Further evidence on the origin of haplotype sharing between GCy and TCy comes from investigating the source of the 24 haplotypes shared between them. This analysis reveals that none of these are found in Turkey (S11 Table), which does not support a Turkish origin of GCy-TCy shared haplotypes. Moreover, 20 out of 24 of these shared haplotypes are absent from the global YHRD database even at the minimal haplotype level, while a considerable proportion include rare alleles, such as unusually small number of repeats, rare duplications and microvariants, suggesting possible local origin (S11 Table).

Pairwise genetic distance (Rst) analysis can reveal shared ancestry beyond haplotype sharing, by simultaneously comparing two sets of haplotypes and determining the proportion of genetic diversity between them. This analysis has revealed a very small statistically non-significant Rst between GCy and TCy indicating primarily a common genetic origin (i.e., belonging to a single panmictic gene pool). Our approach of estimating pairwise genetic distance has been validated by comparing our sample to that from another GCy study[12], which also revealed a very small statistically non-significant Rst. It should be mentioned that more emphasis should be given to the non-significant P-value (evidence of panmictic populations) rather than the actual Rst values, since any small differences in Rst may be due to sampling variation. This observation thus further strengthens the notion of a common paternal ancestry between GCy and TCy (scenario 1). The very low genetic differentiation between GCy and TCy observed in the current study is in agreement to a recent publication on the ancestry of the TCy community, which again shows that among a series of surrounding populations, TCy show the smallest genetic differentiation to GCy (Fst = 0.0091)[14]. A limitation of the specific study however is that it has used Y-haplogroup data of relatively low resolution from previous studies[10,13] and in addition, the exact ethnic origin (i.e. GCy or TCy) of the Cypriot samples from those studies has not been specified. In contrast, our analysis using samples with a very clearly defined and confirmed ethnic origin and analyzing high resolution Y-STR data, has systematically compared GCy and TCy and proved their very close genetic affinity, which in fact was also partly suggested by the aforementioned study[14].

Also, our MJN analysis revealed a population expansion for two clusters within two major haplogroups (E-M123 and E-M78) which happened the earliest ~3500 years ago for the former and ~400 years ago for the latter. The population expansion in the former haplogroup appears to have occurred during late Bronze Age / early Iron Age, while for the latter the expansion could overlap the end of the Venetian era and start of the Ottoman era in Cyprus. Overall, the networks do not support differential mutations in the two populations, indicating that GCy and TCy haplotypes expanded together at a large extent, but at the same time this analysis cannot prove that one population is derived from the other.

In addition to the STR-based analysis and the determination of recent shared ancestry, classification of haplotypes into major Y-haplogroups can help infer ancestral genetic origins and differentiate populations based on their current paternal genetic structure. This analysis is particularly sensitive in detecting influx from distant populations (i.e. with different Y-haplogroup composition) into a genetically different receiver population. Therefore, this analysis is particularly useful in detecting Eastern Eurasian haplogroups (of probable proto-Turkic origin) in the Cypriot gene pool. It should be noted here that due to these different methodological approaches between Y-STR based and Y-haplogroup based analyses, some differences regarding genetic population proximities are not unexpected

The current study is in agreement and confirm results from previous studies[10,12,13] supporting the notion that the major Y-Haplogroups among GCy are J2a and E1b1b (E-M78 and E-M123), followed by G2a and R1b. In particular, our Y-Haplogroup frequencies are in high agreement with those of a recently published study[12], which included a detailed Y-SNP analysis among a sample of GCy (N = 631). In fact after combining our GCy sample with the previously published GCy sample and repeating our analyses, the results were identical. The aforementioned study aimed specifically at testing for deep haplogroup subclades among Cypriots, which helped investigate Neolithic and Bronze Age migrations into the island, but did not include a TCy sample, thus a direct comparison between GCy and TCy in terms of haplogroup frequencies was not made.

In contrast, our study explicitly aimed to systematically compare the paternal ancestry of GCy and TCy and in addition analyzed data in a way as to concentrate on more recent ancestry (past millennium), rather than prehistory. These are the two main novelties of the current study, which complement the results from the aforementioned studies to provide a complete picture of Cypriot paternal ancestry, from prehistory up to modern times.

Our reanalysis of the publicly available TCy data[14], is in agreement with the original study as regards TCy Y-Haplogroup frequencies, with only very few minor differences in haplogroup assignments, resulting from slightly different methodologies followed in haplogroup assignment (for details refer to S3 Table). It should be noted that the main reason for reanalyzing the TCy Y-STR data for Y-Haplogroup determination, was to avoid bias in our comparison with the GCy sample and this was achieved by following identical procedures for *in silico* predicting Y-haplogroups in the two populations (see Methods section for details). Both our analysis and the original analysis by Gurkan et al, have determined that among TCy (as in GCy) the major Y-Haplogroups are J2a and E1b1b. Overall, GCy and TCy were found to have very similar distributions of the major Y-haplogroups.

Haplogroup J2a is predominant in both GCy (23.8%) and TCy (20.3%). There are several speculations regarding the origin of this haplogroup, but recent DNA data on ancient samples revealed that it possibly spread from the Caucasus and modern day Iran to Anatolia and Greece and from there gradually to Cyprus during the Bronze Age[12,41–43].

Haplogroup E-M78 also was found at relatively high frequencies among Cypriots (12.8% GCy, 13.9% TCy). Haplogroup E-M78 is particularly high among Greeks (as well as Albanians) and Cypriot E-M78 belongs predominantly to subclade E-V13 (~10% GCy, ~8% among TCy) (S6 Table), as the majority of Balkanic E-M78[44]. The presence of this haplogroup at similar frequencies within GCy and TCy, much higher than among Turks, strengthens the scenario of TCy being derived from the gene pool of GCy (shared common ancestry). Interestingly, the only region of Turkey with a considerable frequency of E-M78 (8%) is West Anatolia, which is bordering Northeast Greece (Thrace), which itself has a frequency of 17%.

The other major common haplogroup is G2-P287 (12.5% in GCy, 13.7% in TCy), which is believed to have been the major haplogroup brought by Near Eastern (mainly Anatolian) Neolithic farmers to Greece and the Balkans and then to Central/Western Europe[43,45,46]. The presence of G2a in Cyprus in high frequencies is compatible with an early Pre-Pottery Neolithic colonization of Cyprus as supported by recent archaeological findings and ancient DNA data [1,47].

Concentrating on differences in haplogroup frequencies between GCy and TCy, what stands out in qualitative rather than quantitative terms, is the presence of Eastern Eurasian haplogroups (H, C, N, O, Q) at a moderate frequency (~5.5%) in TCy but not in GCy. These haplogroups are prevalent among mainland Turks (ranging from 3% in South Anatolia to 15% in Central Anatolia) (Fig 3; S4 and S5 Figs). The Central Asian origin[37,38,48–51] of some of these haplogroups, namely C, N, and Q, points to the influx of proto-Turkic tribes in the Anatolian peninsula, establishing gradually the Ottoman Empire and spreading to Cyprus during the Ottoman era (1571–1878), to be assimilated into the TCy gene pool. In fact, the current findings indicate that the frequency of these possible proto-Turkic haplogroups among TCy is 4.2% (S8 Table). In addition, TCy show evidence of low frequencies of North African haplogroups (E-M81, E-V38). The fact that these haplogroups are absent from GCy may suggest a minor influx of North African males to Cyprus, either assimilated within the Ottoman settlers (civilians and soldiers) or brought as slaves, a common practice during the Ottoman era, who were apparently gradually assimilated into the TCy community[52].

TCy show lower frequencies of haplogroups E-M123 and R1b compared to GCy (6.3% vs 13.1% and 11.9% vs. 6.8%). E-M123 is found at very low frequencies among both mainland Greeks and Turks (apart from South Turks, 9%). Recently this haplogroup has been found in human remains from Bronze Age Armenia[53], while a subclade ancestral to E-M123 (E-PF1961/Z830) has been found among Natufian cultures from Epipaleolithic Israel[54] pointing to this being a South Levantine Neolithic haplogroup, probably already present in Cyprus from prehistoric years. R1b is a haplogroup has been shown through analysis of ancient DNA samples to be highly prevalent among Bronze Age Steppe cultures (e.g. the Yamnaya)[55], as well as Mesolthic Balkan populations[56] and Bell-Beaker cultures[57]. R1b might have entered Cyprus from Greece, Anatolia, or even the Levant as indicated by a previous study[12]. It is possible, that for both E-M123 and R1b, the differences in frequencies between GCs and TCs were brought about randomly, as a consequence of differential conversion rates (from Christianity to Islam) in the different regions of Cyprus, where the different haplogroups are differentially distributed[12]. In addition, there might have been a ‘dilution’ effect resulting from recent admixture in TCy with populations low in E-M123 and R1b (e.g. some Turkish sub-populations and North Africans). The above scenario is further supported by the presence of shared haplotypes between TCy and mainland Turkish and North African populations (see subsequent sub-sections) (Fig 1). Of interest, is also a difference in the frequency of haplogroup I2 (2.3% aming GCy, 6.8% among TCy), which may indicate a minor influx of Balkanic individuals, along the Ottoman settlers, who possibly served in the Ottoman army (e.g. janissaries)[58].

To sum up, all analyses performed in the current study point to a primarily common paternal ancestry between GCy and TCy, despite some differences in current Y-haplogroup distribution, which might indicate differential admixture with surrounding populations over the past few centuries (see subsequent sub-section).

Additional evidence, that further supports a common ancestry between GCy and TCy[59], comes from a study showing that in Cyprus four mutations were responsible for the majority of beta-thalassaemia cases (>79%). While similar frequencies of these mutations were observed between TCy and GCy, much lower frequencies were present in patients from Turkey and Greece.

### Shared paternal ancestry between Cypriots and Greeks

Previous evidence on the paternal ancestry of GCy based on detailed SNP data, revealed that approximately 13% of Cypriot patrilinages have a Balkanic origin, characterized primarily by haplogroups E-V13 and I2, as well as specific sub-clades of G2a, introduced in the island from mainland Greece during the late Bronze Age and throughout the Iron Age[12].

In the current study, haplotype sharing between GCy and Greeks is in the range of 1.5%, (much lower than between GCy and TCy, 7–8%). Haplotype sharing between TCy and Greeks is somewhat lower, in the range of ~1%. The low haplotype sharing between Greeks and Greek Cypriots is not surprising, as the major Greek migrations to Cyprus (described in the aforementioned study), occurred 2–3 millennia ago, while shared haplotypes in our analysis indicate common ancestry of around 1000 years or less. Therefore, these results indicate that in the past 1000 years, there has been very little gene flow (at least paternally) from Greece to the GCy population.

In terms of Rst pairwise genetic differences, indicating deeper shared paternal ancestry than the shared haplotype analysis, Greeks appear genetically close to Cypriots (the closest population after the Lebanese and Calabrian Italians). In fact, Greeks show similar differentiation from both GCy and TCy (i.e., they are equally distant from them), which, further supports scenario 1 (common local ancestry of GCy and TCy) rather than scenario 2 (recent non-local ancestry of TCy and subsequent intermixing with GCy). Similar, to the current findings, a previous study on the paternal ancestry of TCy[14] also showed that the genetic differentiation between TCy and Greeks and TCy and Turks is similar (Fst = 0.0215 and Fst = 0.0226, respectively). It should be noted here that Rst distances based on Y-STR data (current study) and Fst distances based on Y-haplogroup data (previous TCy study)[14] are not directly comparable in absolute terms.

In terms of Y-haplogroup distribution, Cypriots (GCy and TCy) show substantial differences from Greeks, characterized by much lower frequency of haplogroups I2, R1a, and R1b in the former. These haplogroup differences indicate differential migrations into Cyprus and mainland Greece, at different points in history and prehistory. I2 is considered the major haplogroup among Mesolithic European Hunter-Gatherers[60], who apparently were either absent from Cyprus or were totally diluted (nearly extinguished) by subsequent migrations. Although the exact origins and migratory patterns of R1a and R1b are still under rigorous investigation, it seems that they are linked to Bronze Age migrations from the Western Eurasian Steppe and Eastern Europe into Southern (including Greece) and Western Europe[61]. Apparently, such migrations (especially as regards R1a) into Cyprus were limited.

Additionally, the Greek population has received considerable migrations during the Byzantine era and the Middle Ages from other Balkanic populations, such as Slavs[62,63], Aromanians (Vlachs)[64], and Albanians (Arvanites)[65,66]. The former, is very likely to have increased R1a frequencies among Greeks. In fact, Fig 3 (also S7 Table) indicate that R1a increases gradually with increasing latitude in Greece. There is no historical evidence for such migrations into Cyprus during the same period.

The only Greek sub-population showing close genetic proximity to Cypriots (in terms of Y-haplogroup composition) is Cretan Greeks (Figs 3 and 4). It could be speculated that Cypriots and Cretans experienced very similar migratory events over the centuries, which were characterized by high influx from populations rich in haplogroups J2a and G2, and moderate in R1b, while very limited influx from populations rich in haplogroups R1a and I (Eastern and Northern/Central Europe), as well as from populations rich in J1 (Middle East) and E-M81 (North Africa).

It should be noted here that the genetic comparisons based on Y-STR haplotypes conducted in the current study between Cypriots and Greeks cannot be considered exhaustive, since the Greek population is not well represented in terms of Y-STR haplotypes, with important regions in close proximity to Cyprus, such as Crete and the Aegean islands missing. Given the high similarity in Y-haplogroup frequencies between Cypriots and Cretan Greeks it is likely that comparisons at the Y-STR level would reveal a high genetic affinity between these populations.

To sum up, the current study confirms previous findings of a relatively high frequency of haplogroup E-V13 among Cypriots (both GCy and TCy)[12,44], which points possibly to an influx of Aegean populations into Cyprus during the late Bronze Age / early Iron Age. Our analysis further reveals a relatively high genetic affinity between Greeks and Cypriots (both GCy and TCy) based on Y-STR analysis, but very little admixture between the two populations during the past millennium.

### Shared paternal ancestry between Cypriots and Turks

Previous evidence using deep haplogroup subclade data, revealed that approximately 66% of Cypriot patrilinages have an Anatolian origin, characterized primarily by haplogroups G2a, J2a, and R1b, introduced in the island throughout the Neolithic (former) and Bronze Age (latter two; particularly early Bronze Age i.e. Philia culture)[12].

In the current study, no shared haplotypes were observed between GCy and modern Turks It should be noted that this finding is not in contradiction to the aforementioned finding of substantial genetic contribution from Neolithic/Bronze Age Anatolia to Cyprus. There are two main reasons for this. Firstly, haplotype sharing at the level presented in the current study (17/17 Y-STRs) can only be relevant to shared ancestry of the past few centuries (past 1000 years at the max). Neolithic and Bronze Age migrations cannot be reflected in such analysis since any shared haplotypes surely diverged and differentiated substantially during the millennia. In addition, although some degree of genetic continuity could be expected in Anatolia (i.e. in modern Turks), it should be noted that modern Turks are a hybrid population, comprising of the original Anatolian stock, Turkic people (i.e. of Central Asian ancestry), as well as other ethnicities from regions comprising the former Ottoman Empire. This is surely reflected in the modern Turkish Y-DNA and thus any genetic deviations between Cypriots and Turks (current study) do not defy the substantial Neolithic/Bronze Age migrations from Anatolia to Cyprus, previously discussed[12]. In fact, our PCA analysis clearly indicates clustering of GCy with non-Turkic Anatolian populations (Kurds and Armenians), as well as South Turks (showing particularly low frequencies of Eastern Eurasian haplogroups, S7 Table), which may indicate a common deep paternal ancestry characterized of population movements during the Neolithic and Bronze Age.

Concentrating to more recent history, the lack of shared haplotypes observed between GCy and Turks in the current study, indicates an apparently null (or extremely limited) penetration of Turkish paternal haplotypes into the GCy gene pool, despite 300 years of Ottoman rule of the island. In contrast, TCy show clear evidence of haplotype sharing with Turks (~3% of individuals carrying TCy haplotypes). The finding that TCy share haplotypes with Turks indicates at least a partial paternal origin from mainland Turkey, but the moderate genetic differentiation (Rst) between TCy and Turks (Table 2) does not support the notion that TCy primarily derive from the same paternal gene pool as mainland Turks (scenario 2 in Introduction). However, a previous study on the paternal ancestry of TCy[14] showed somewhat lower genetic differentiation between TCy and specific geographical sub-groups of Turks (e.g. East, Southeast, Mediterranean, and Marmara regions). This analysis however is based on lower resolution Y-STR data and smaller population samples. Our analysis using the online YHRD AMOVA tool on higher resolution Y-STRs, did confirm a low genetic differentiation between TCy and specific Turkish sub-populations (e.g. TCy vs East Anatolia), which is not very different than the differentiation observed between TCy and North Greeks and smaller than the differentiation observed between TCy and GCy in the same analysis (Table 2), thus in agreement with our study.

In terms of Y-haplogroup analysis, a major feature differentiating Turks from Cypriots (particularly GCy), is the presence of Eastern Eurasian haplogroups in the former ranging from 3% (South Turks) to 15% (Central Turks). TCy, with a frequency of these lineages ~5.5%, fall in the lower margins of the range of frequencies of mainland Turks. In fact, in the Eastern Mediterranean / Near East region (with the exception of Anatolian Armenians from Sasun), Eastern Eurasian haplogroups are found in considerable frequencies only among Turkish populations (S7 Table, S6 and S7 Figs). Another differentiating characteristic of both Cypriot groups from Turks is the higher frequency of haplogroup E-M78 among the former.

Our PCA plot (Fig 4) indicates a clear separation of Turkish sub-populations away from Cypriots, Southeast European and other Near Eastern and North African populations. TCy, although sharing some similar features to mainland Turks (i.e. presence of Eastern Eurasian haplogroups) do not seem to cluster with Turkish sub-populations in the PCA plot (Fig 4A). When Eastern Eurasian haplotypes were replaced in the TCy sample (see Methods, as well as S8 Table), the TCy shifted more towards GCy in the plot and and further away from the Turkish populations (Fig 4B).

To sum up, the current study confirms previous findings of a moderate genetic affinity, based on Y-STR analysis, between Turks and Cypriots (both GCy and TCy)[14], Our analysis also confirms the presence of relatively high frequencies of haplogroup G2a and particularly J2a, both of which suggested to have arrived to Cyprus primarily from Anatolia during the Neolithic and Bronze Age, respectively. Our analysis further reveals substantial recent admixture between Turks and Turkish Cypriots, proving thus a considerable contribution of mainland Turks in the Turkish Cypriot community during the Ottoman era.

### Shared paternal ancestry between Cypriots and other populations

Overall, there is very minor contribution to the paternal Cypriot gene pool from surrounding populations within the past millennium, as indicated by the limited extent of haplotype sharing. Populations other than Greeks and Turks, from the Southeast Europe, the Near East and North Africa, which appear to share considerable (yet small) numbers of haplotypes with Cypriots are: Italians and Albanians (particularly with GCy), Lebanese (both GCy and TCy), and Libyans (only TCy).

Despite limited haplotype sharing, a population shows remarkable genetic affinity with Cypriots (both GCy and TCy), with evidence of non-significant genetic differentiation, indicating shared paternal ancestry in the more distant past; namely Calabrian Italians. It should be noted however that the Calabrian samples used in the current analysis were relatively small (n = 30 comparative dataset, n = 74 YHRD) and thus these results should be interpreted with caution.

If the high genetic affinity observed between Cypriots and Calabrian Italians is assumed to be true, it could be explained by the fact that South Italy has been a part of the ancient Greek world for centuries (Magna Graecia) and Calabria in particular has been settled by Achaean Greeks during the 8th and 7th cent. B.C [as Cyprus was, a few centuries back[67]]. Thus the high genetic affinity between Calabrians and Cypriots could be a result of a common ancient Greek (Achaean) genetic contribution to both populations.

Lebanese patrilinages also appear very close to Cypriot (both GCy and TCy).patrilinages in terms of paternal ancestry. The high genetic affinity between Cypriots and Lebanese can be explained through several migrations that took place from coastal Levant to Cyprus from the Neolithic (early farmers)[1] to the Iron Age (Phoenicians)[3] and up to the Middle Ages (Maronites and other Levantine settlers during the Frankish era)[4]. A previous study analyzing detailed SNP data for determining ancient ancestry among Cypriots, revealed that around 24% of Cypriot patrilinages are descendent from the Levant, derived from migrations occurring from the Neolithic and throughout the Bronze and Iron Ages[12].

The fact that both GCy and TCy show very similar (high) genetic affinity with these two populations, highlights again a very possible common ancestry for the two Cypriot communities. In fact, the above results combined indicate that, Cypriots are placed in the middle of an apparent genetic continuity extending from the Near East to Southeastern Europe.

However, despite the very low genetic differentiation between Cypriots, Calabrian Italians, and Lebanese, the former appear to differentiate, in terms of Y-haplogroup frequencies, both from Middle Eastern (including Lebanese) and from Southeast European Mediterranean (including South Italians) populations. The main feature distinguishing Cypriots from Lebanese and other Middle Easterners included in our analysis is their much lower frequency of haplogroup J1. This observation clearly suggests that although Cypriots and Lebanese share common paternal roots, the latter received a substantial influx from populations high in J1, probably during the early Arab conquest era (7^th^ cent. AD). Similarly, North Africans also are particularly high in haplogroup E-M81, which is extremely rare (TCy) or absent (GCy) in Cyprus.

The separation of Cypriots from Southeast European Mediterranean populations included in our analysis is brought about by the much lower frequency in the former of haplogroups I2, R1a and R1b. South Italians in particular, although relatively low in haplogroups I2 and R1a, have a substantial proportion of haplogroup R1b (Fig 3). This difference suggests that although Calabrian Italians share primarily common paternal genetic roots with Cypriots, there has been an influx of populations high in R1b, which affected South Italy much more than Cyprus. With the lack of ancient DNA data from either region, it is difficult to disentangle the origins of this differentiation.

GCy do show Y-haplogroup clustering with non-Turkic Anatolian and Southwest Caucasian populations (Anatolian Kurds and specific Armenian groups) (Fig 4). These similarities may have been brought about by common distant migratory events, but cannot prove recent shared ancestry, which is better demonstrated by the amount of shared haplotypes and the Rst differentiation results discussed above. These analyses revealed no haplotype sharing and relatively high differentiation between Anatolian Kurds and Cypriots, indicating that any similarities in haplogroup frequencies between the two are likely not due to recent shared ancestry but more a result of distant migratory events. Unfortunately, Y-STR haplotype data for Armenians are missing from the literature. The Armenians have a continuous presence in Cyprus since the 6^th^ century AD, with a well-documented Armenian community up to this day [68,69]. With a lack of Armenian Y STR-haplotype data, it is not possible to prove any recent shared paternal ancestry.

### Historical context

From a historical perspective, throughout the centuries, the island’s demographic ratio between Greek Orthodox Christians and Muslim Turks varied. Crypto-Christians were individuals who, in despair, were forced to convert to another religion. They did not fully denounce their faith; rather, practiced it in secret. In Cyprus, such individuals were known as ‘*Linobambaki*’ from the Greek ‘*Λινοβάμβακοι*’[7] and records suggest GCy did convert to Islam at different periods[4,70]. According to the Greek Consul’s Report of 1869, the numbers of Linobambaki were stated as 10,000–15,000. Once Cyprus became part of the British Empire, the majority of the Linobambaki reintegrated into the Christian Orthodox community of Cyprus while the remaining maintained a permanent Muslim status[71] (i.e., assimilated in the current TCy community). Thus, there is support to a common genetic background of the two communities.

On the other hand, historical evidence also suggests a population influx (estimated 10,000 Ottoman soldiers initially and later followed by civilians) from Anatolia, as well as other Ottoman regions in the South Caucasus, Central Asia, and North Africa to Cyprus from the beginning of the Ottoman era (1571) and throughout the 3 centuries of Ottoman domination that follow[58]. Therefore, these events can explain the moderate numbers of shared haplotypes found between TCy and mainland Turks, as well as the presence of Eastern Eurasian and North African haplogroups. Regarding the relatively high frequency of Eastern Eurasian haplogroups (~5.5%) among TCy, which is not much lower than those observed among mainland Turks (3–15%), it should be noted that some of the original Ottoman solders (e.g. the Azabs, the Sekban, and the Akinci) did not originate from Anatolia, but from other regions of the Ottoman Empire, including regions of the Caucasus and Central Asia, comprising of ethnic groups such as Tatars, Nogay, and Turkmen, which could provide a potential explanation for this observation. Another potential explanation could be that the Ottoman settlers of the 16th century and their ancestors, were particularly successful in passing on their genes (i.e. had a relatively high number of offspring), which would then lead to some kind of genetic drift, increasing thus the frequency of Eastern Eurasian haplogroups. In fact, we do observe in the current study, that haplotypes shared between Turkish Cypriots and Turks were more likely to be found in duplicate and triplicate (i.e. shared by two or three unrelated individuals) in the Turkish Cypriot sample (S11 Table).

In conclusion, our Y-chromosome analyses reveal that in terms of paternal linages, GCy share primarily a common paternal ancestry with TCy, which based on the current findings, is of local origin. Moreover, the latter show evidence of recent (past few centuries) genetic contribution from mainland Turkey and presence of minor Eastern Eurasian and North African paternal ancestry.

## Supporting information

S1 Fig Locations in the Central/Eastern Mediterranean and Near East from which detailed Y-haplogroup data were derived from the literature and compared to the Greek Cypriot sample. A list of all populations and sub-populations included is presented in S5 Table.
(TIF)

S2 Fig **Contour maps displaying Rst distances between (a) Greek Cypriots and (b) Turkish Cypriots and other Western Eurasian and North African populations.** The intensity of the colour in the contour maps corresponds to the magnitude of the Rst distance (darker colour indicates smaller Rst, which in turn indicates low genetic differentiation between populations). The colour intensity in the island of Cyprus represents the size of the Rst between Greek Cypriots and Turkish Cypriots.
(TIF)

S3 Fig **Median-joining networks for haplogroups (a) J2a, (b) J1, (c) E-M78, (d) E-M123, (e) G2a, and (f) R1b.** Median Joining Network based on 12 Y-STR loci. For each network, blue colour indicates GCy haplotypes and red colour TCy haplotypes. Circles are sized according to the number of individuals sharing the haplotype, with the smallest circles representing one individual. The lengths of the connecting lines are proportional to the number of mutational steps separating two haplotypes.
(TIF)

S4 Fig **Median-joining networks for haplogroups (a) J2a, (b) J1, (c) E-M78, (d) E-M123, (e) G2a, and (f) R1b.** Median Joining Network based on 17 Y-STR loci. For each network, blue colour indicates GCy haplotypes, after combining the current sample with a previously published GCy sample. and red colour indicates TCy haplotypes. Circles are sized according to the number of individuals sharing the haplotype, with the smallest circles representing one individual. The lengths of the connecting lines are proportional to the number of mutational steps separating two haplotypes.
(TIF)

S5 Fig Y-haplogroup frequencies among Greek Cypriots, Turkish Cypriots and populations from the Central and Eastern Mediterranean and Near East regions. Population codes as in S5 Table.
(TIF)

S6 Fig Frequencies of Western Eurasian (light blue), Eastern Eurasian (purple) and North African (brown) Y-haplogroups among Greek Cypriots, Turkish Cypriots, Greeks and Turks. Y-haplogroups were geographically classified according to current distribution among modern populations rather than possible ancestral haplogroup origin. Western Eurasian Y-Haplogroups: E1b1b (M78, M123), G1, G2, I1, I2, J1, J2, K, L, R1a, R1b, T. Eastern Eurasian Y-Haplogroups: C, H, N, O, Q, R2. North African Y-Haplogroups: A, B, DE, E1a, E1b1*, E1b1a, E1b1b (M81).
(TIF)

S7 Fig Frequencies of Western Eurasian (light blue), Eastern Eurasian (purple) and North African (brown) Y-haplogroups among Greek Cypriots, Turkish Cypriots, and populations from the Central/Eastern Mediterranean and Near East regions. Y-haplogroups were geographically classified as described in S6 Fig.
(TIF)

S1 Table Y-STR haplotype data for the Greek Cypriot population and Y-haplogroup assignment. (XLSX)

S2 Table SNPs used including primer sequences and detection method (XLSX)

S3 Table Y-STR haplotype Data for the Turkish Cypriot Population and Y-haplogroup Assignment (XLSX)

S4 Table Details of the Y-STR dataset used for the pairwise genetic distance analysis and the shared haplotype analysis. (DOCX)

S5 Table Details of the Y-Haplogroup based dataset used in the analysis. (XLSX)

S6 Table Y-haplogroup and major subclade frequencies among Greek Cypriots and Turkish Cypriots. (XLSX)

S7 Table Y-haplogroup frequencies among Cypriots and surrounding populations. (XLSX)

S8 Table Y-haplogroup distribution among Greek Cypriots and Turkish Cypriots representing the actual current distribution and the predicted pre-Ottoman distribution following exclusion of possible proto-Turkic Y-haplogroups (XLSX)

S9 Table Haplotype diversity indices for Cypriot, Greek and Turkish samples included in the comparative dataset (XLSX)

S10 Table Shared haplotypes between Cypriots and other Western Eurasian and North African populations (XLSX)

S11 Table Haplotypes shared between Greek Cypriots, Turkish Cypriots, Greeks, and Turks, including Y-haplogroup assignment (XLSX)

S12 Table Pairwise genetic distances (Rst values) between Greek Cypriots, Turkish Cypriots, Greeks, Turks, and other Western Eurasian and North African populations included in the comparative dataset (XLSX)
