# Supplementary figures and images for: Y-chromosomal analysis of Greek Cypriots reveals a primarily common pre-Ottoman paternal ancestry with Turkish Cypriots
Source: PLoS One. 2017 Jun 16;12(6):e0179474. doi: 10.1371/journal.pone.0179474 (PMC5473566; doi:10.1371/journal.pone.0179474)

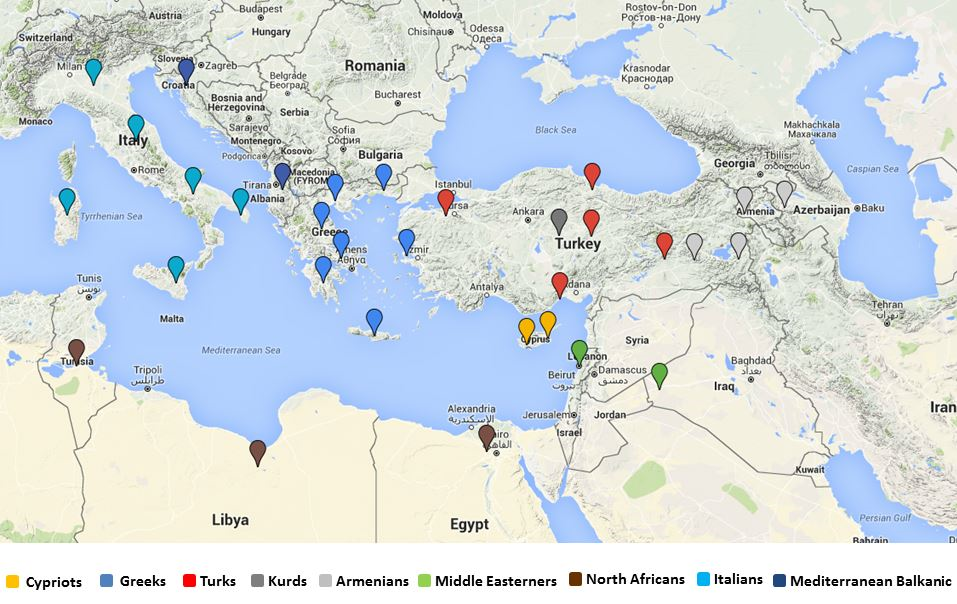

Supplement: S1 Fig — A list of all populations and sub-populations included is presented in S5 Table. (TIF) [file pone.0179474.s001.tif]

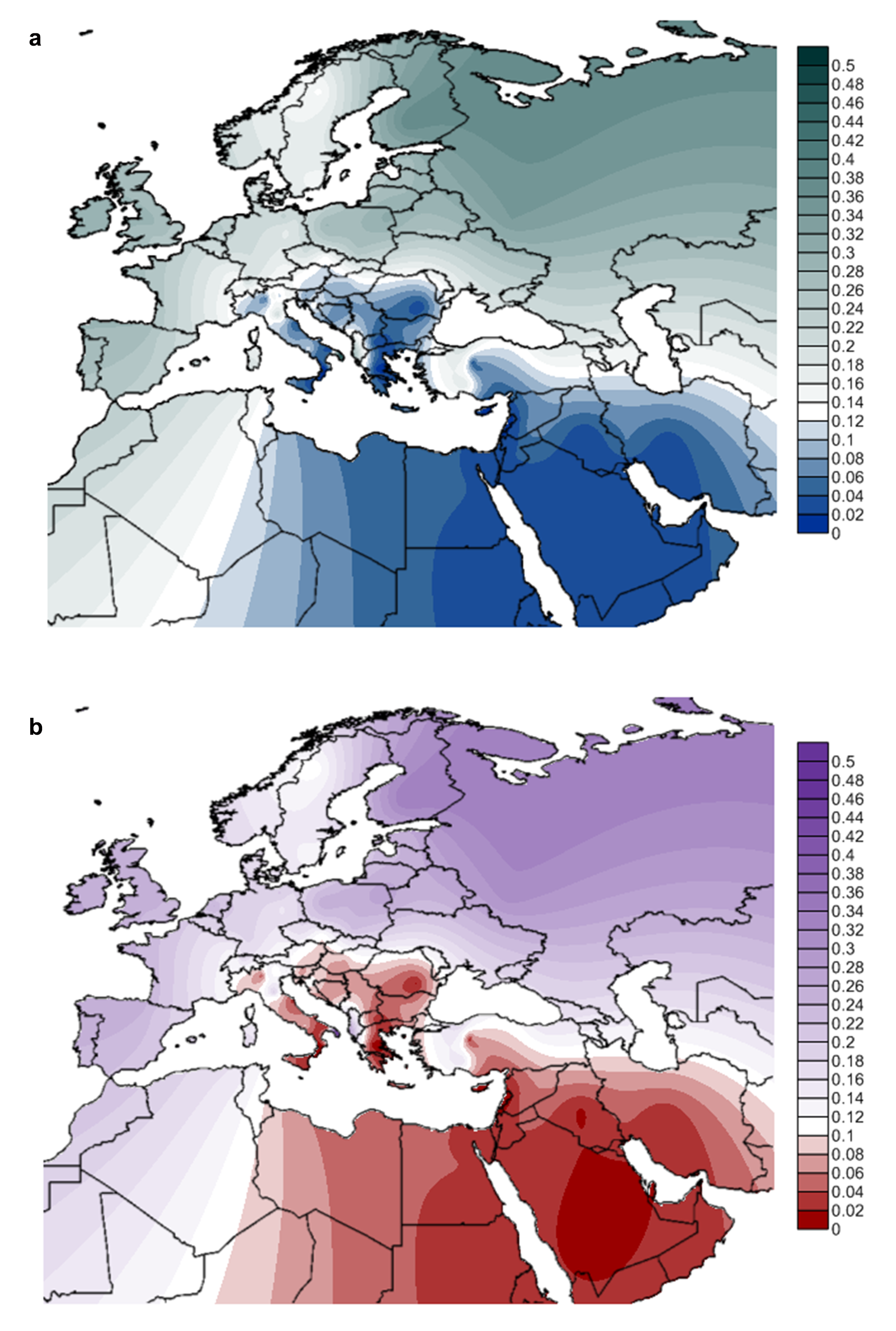

Supplement: S2 Fig — Contour maps displaying Rst distances between (a) Greek Cypriots and (b) Turkish Cypriots and other Western Eurasian and North African populations. The intensity of the colour in the contour maps corresponds to the magnitude of the Rst distance (darker colour indicates smaller Rst, which in turn indicates low genetic differentiation between populations). The colour intensity in the island of Cyprus represents the size of the Rst between Greek Cypriots and Turkish Cypriots. (TIF) [file pone.0179474.s002.tif]

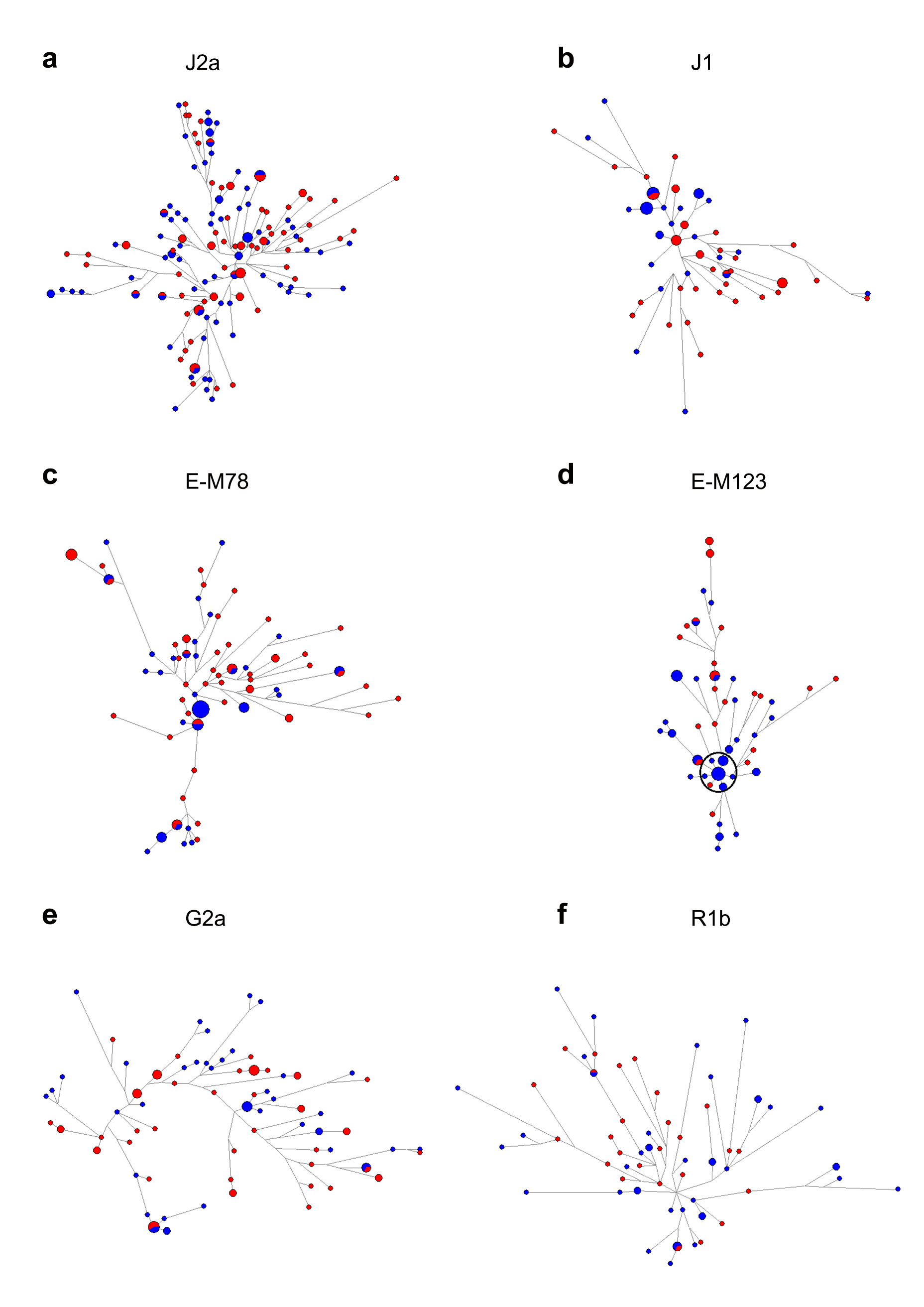

Supplement: S3 Fig — Median-joining networks for haplogroups (a) J2a, (b) J1, (c) E-M78, (d) E-M123, (e) G2a, and (f) R1b. Median Joining Network based on 12 Y-STR loci. For each network, blue colour indicates GCy haplotypes and red colour TCy haplotypes. Circles are sized according to the number of individuals sharing the haplotype, with the smallest circles representing one individual. The lengths of the connecting lines are proportional to the number of mutational steps separating two haplotypes. (TIF) [file pone.0179474.s003.tif]

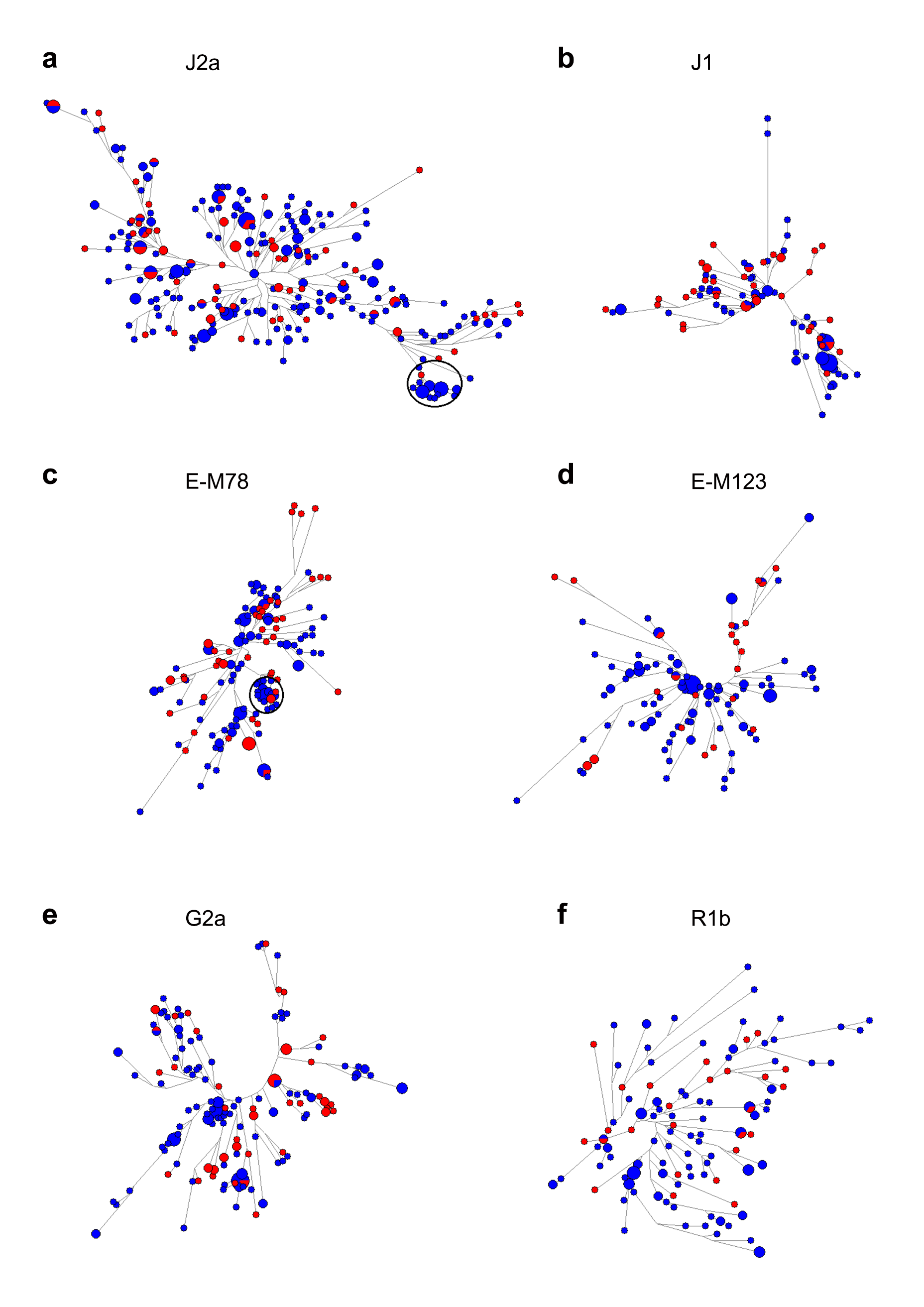

Supplement: S4 Fig — Median-joining networks for haplogroups (a) J2a, (b) J1, (c) E-M78, (d) E-M123, (e) G2a, and (f) R1b. Median Joining Network based on 17 Y-STR loci. For each network, blue colour indicates GCy haplotypes, after combining the current sample with a previously published GCy sample. and red colour indicates TCy haplotypes. Circles are sized according to the number of individuals sharing the haplotype, with the smallest circles representing one individual. The lengths of the connecting lines are proportional to the number of mutational steps separating two haplotypes. (TIF) [file pone.0179474.s004.tif]

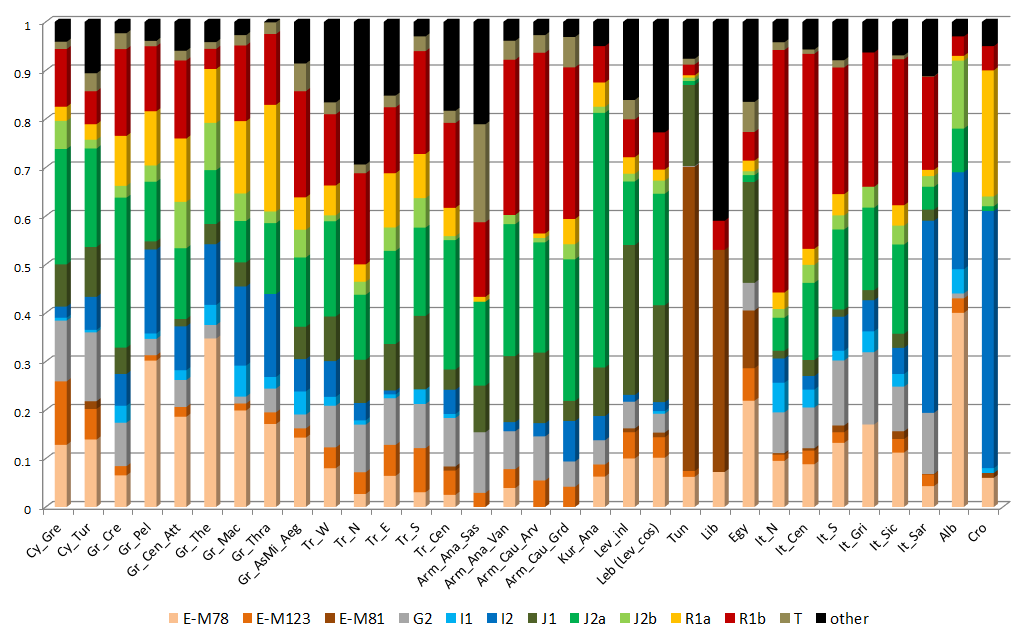

Supplement: S5 Fig — Population codes as in S5 Table. (TIF) [file pone.0179474.s005.tif]

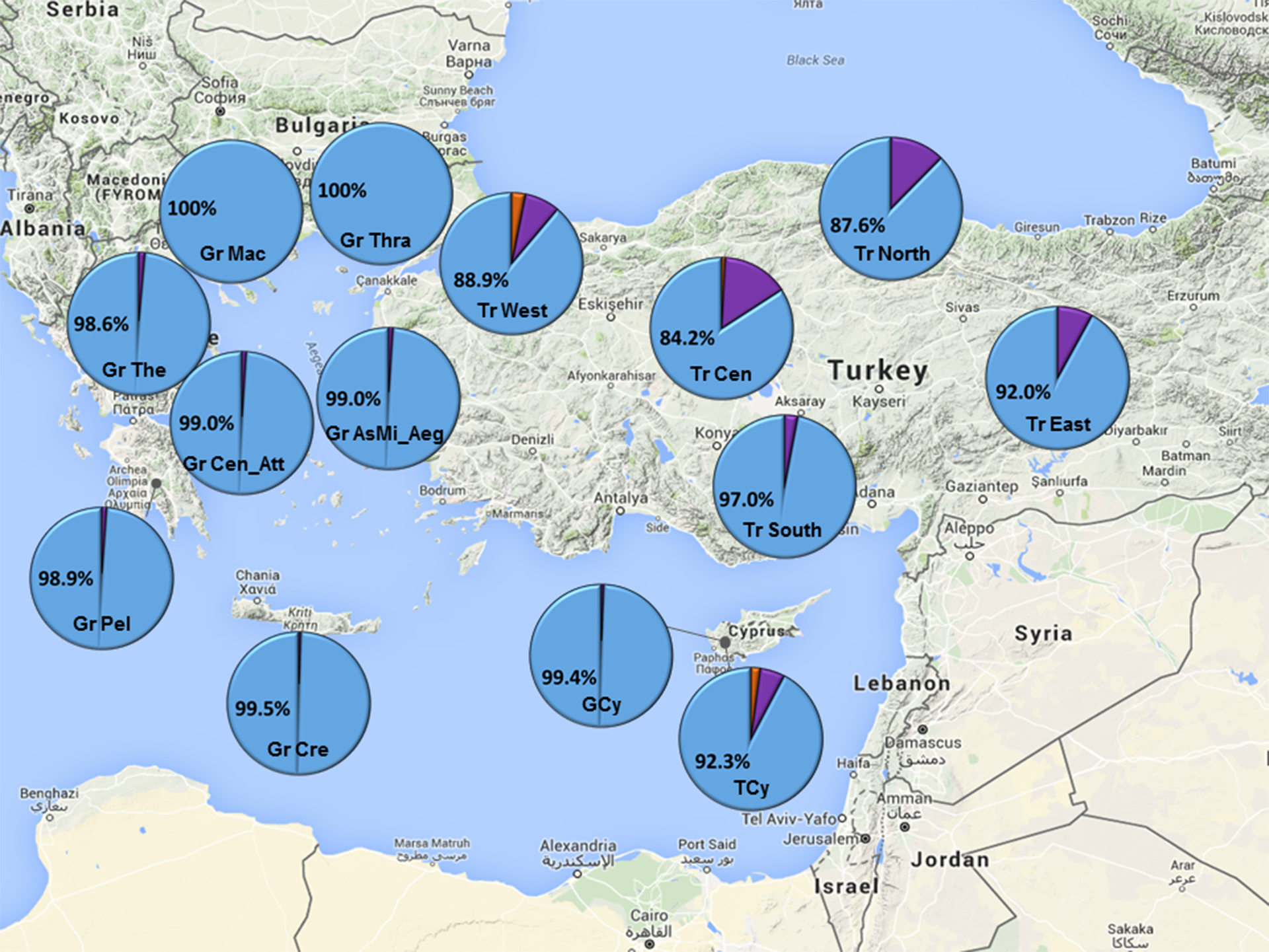

Supplement: S6 Fig — Y-haplogroups were geographically classified according to current distribution among modern populations rather than possible ancestral haplogroup origin. Western Eurasian Y-Haplogroups: E1b1b (M78, M123), G1, G2, I1, I2, J1, J2, K, L, R1a, R1b, T. Eastern Eurasian Y-Haplogroups: C, H, N, O, Q, R2. North African Y-Haplogroups: A, B, DE, E1a, E1b1*, E1b1a, E1b1b (M81). (TIF) [file pone.0179474.s006.tif]

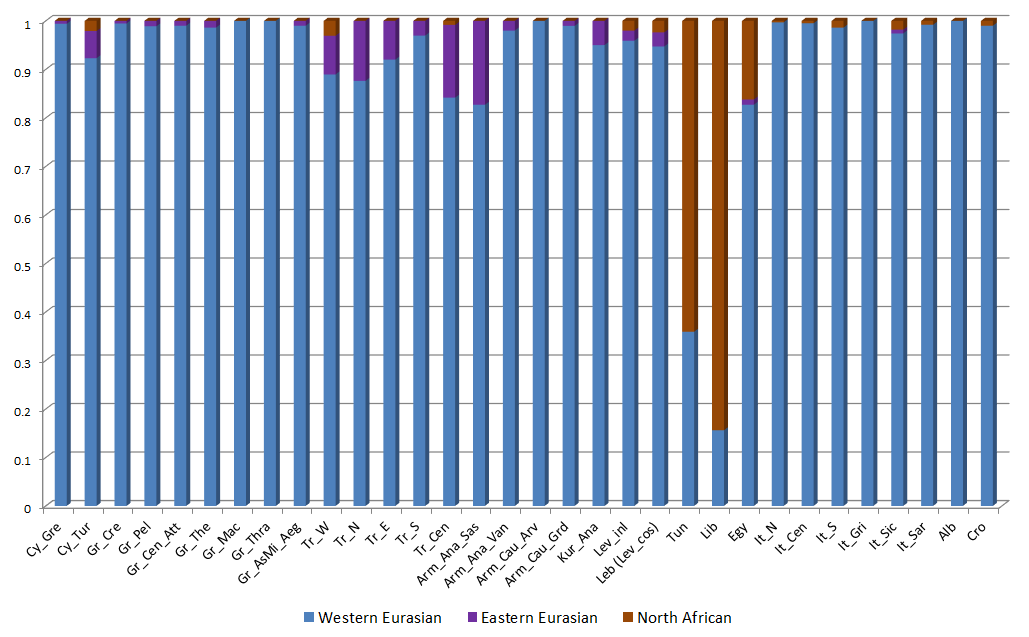

Supplement: S7 Fig — Y-haplogroups were geographically classified as described in S6 Fig. (TIF) [file pone.0179474.s007.tif]
