# Supplementary material for: Y-chromosomal analysis of Greek Cypriots reveals a primarily common pre-Ottoman paternal ancestry with Turkish Cypriots
Source: PLoS One. 2017 Jun 16;12(6):e0179474. doi: 10.1371/journal.pone.0179474 (PMC5473566; doi:10.1371/journal.pone.0179474)
Supplement: S4 Table — (DOCX) [file pone.0179474.s011.docx]

| **S4 Table. Details of the Y-STR dataset used for the pairwise genetic distance analysis and the shared haplotype analysis** | | | | | | | |
| --- | --- | --- | --- | --- | --- | --- | --- |
| **Database** | **Country** | **City/Region** | **Ethnic affinity** | **N database** | **N Rst** | **Contour map** | **Reference** |
| YHRD | Afghanistan | Nationwide | Pathan | 125 | 125 | No | YP000747 (http://yhrd.org - Release 49, 2015/02/17) |
| YHRD | Afghanistan | Nationwide | Afghan | 169 | 152 | No | YP000877 (http://yhrd.org - Release 49, 2015/02/17) |
| YHRD | Afghanistan | North Afghanistan | Pathan | 44 | 44 | No | YP000749 (http://yhrd.org - Release 49, 2015/02/17) |
| YHRD | Afghanistan | South Afghanistan | Pathan | 145 | 142 | No | YP000748 (http://yhrd.org - Release 49, 2015/02/17) |
| YHRD | Albania | Nationwide | Albanian | 100 | 98 | Yes | YP000004 (http://yhrd.org - Release 49, 2015/02/17) |
| YHRD | Albania | Nationwide | Tosk Albanian | 103 | 101 | No | YP000684 (http://yhrd.org - Release 49, 2015/02/17) |
| YHRD | Albania | Nationwide | Gheg Albanian | 119 | 116 | No | YP000683 (http://yhrd.org - Release 49, 2015/02/17) |
| YHRD | Algeria | Oran | Arab | 102 | 76 | Yes | YP000298 (http://yhrd.org - Release 49, 2015/02/17) |
| YHRD | Austria | Oberöstereich | Austrian | 234 | 224 | Yes | YP000295 (http://yhrd.org - Release 49, 2015/02/17) |
| YHRD | Austria | Reutte | Tyrolean | 259 | 253 | Yes | YP000750 (http://yhrd.org - Release 49, 2015/02/17) |
| YHRD | Austria | Salzburg | Austrian | 202 | 193 | Yes | YP000352 (http://yhrd.org - Release 49, 2015/02/17) |
| YHRD | Austria | East Tyrol | Tyrolean | 270 | 262 | Yes | YP000751 (http://yhrd.org - Release 49, 2015/02/17) |
| YHRD | Azerbaijan | Lenkoran | North Talysh | 47 | 37 | Yes | YP000515 (http://yhrd.org - Release 49, 2015/02/17) |
| YHRD | Belgium | Antwerpen | Belgian | 278 | 270 | Yes | YP000700 (http://yhrd.org - Release 49, 2015/02/17) |
| YHRD | Belgium | Limburg | Belgian | 139 | 136 | Yes | YP000783 (http://yhrd.org - Release 49, 2015/02/17) |
| YHRD | Belgium | Oost-Vlaanderen | Belgian | 162 | 158 | Yes | YP000784 (http://yhrd.org - Release 49, 2015/02/17) |
| YHRD | Belgium | Vlaams-Brabant | Belgian | 158 | 151 | Yes | YP000701 (http://yhrd.org - Release 49, 2015/02/17) |
| YHRD | Belgium | Walloon | Belgian | 47 | 43 | Yes | YP000785 (http://yhrd.org - Release 49, 2015/02/17) |
| YHRD | Belgium | West-Vlaanderen | Belgian | 190 | 188 | Yes | YP000786 (http://yhrd.org - Release 49, 2015/02/17) |
| YHRD | Bosnia and Herzegovina | Nationwide | Bosnian | 100 | 94 | Yes | YP000824 (http://yhrd.org - Release 49, 2015/02/17) |
| YHRD | Bulgaria | Nationwide | Bulgarian | 91 | 91 | Yes | YP000064 (http://yhrd.org - Release 49, 2015/02/17) |
| YHRD | Bulgaria | Nationwide | Romani | 228 | 228 | No | YP000065 (http://yhrd.org - Release 49, 2015/02/17) |
| YHRD | Croatia | Central Croatia | Croatian | 220 | 212 | Yes | YP000624 (http://yhrd.org - Release 49, 2015/02/17) |
| YHRD | Croatia | Nationwide | Croatian | 125 | 119 | Yes | YP000916 (http://yhrd.org - Release 49, 2015/02/17) |
| YHRD | Croatia | East Croatia | Croatian | 220 | 212 | Yes | YP000625 (http://yhrd.org - Release 49, 2015/02/17) |
| YHRD | Croatia | North Croatia | Croatian | 220 | 216 | Yes | YP000626 (http://yhrd.org - Release 49, 2015/02/17) |
| YHRD | Croatia | South Croatia | Croatian | 220 | 218 | Yes | YP000627 (http://yhrd.org - Release 49, 2015/02/17) |
| YHRD | Croatia | West Croatia | Croatian | 220 | 216 | Yes | YP000628 (http://yhrd.org - Release 49, 2015/02/17) |
| YHRD | Croatia | Zagreb | Croatian | 114 | 113 | Yes | YP000472 (http://yhrd.org - Release 49, 2015/02/17) |
| YHRD | Cyprus | Nationwide | Turkish Cypriots | 380 | 328 | Yes | YP000884 (http://yhrd.org - Release 49, 2015/02/17) |
| YHRD | Czech Republic | Bohemia | Czech | 72 | 70 | Yes | YP000804 (http://yhrd.org - Release 49, 2015/02/17) |
| YHRD | Czech Republic | Moravia | Czech | 42 | 39 | Yes | YP000805 (http://yhrd.org - Release 49, 2015/02/17) |
| YHRD | Denmark | Nationwide | Danish | 185 | 182 | Yes | YP000115 (http://yhrd.org - Release 49, 2015/02/17) |
| YHRD | Egypt | Assiut | Egyptian | 70 | 55 | Yes | YP000022 (http://yhrd.org - Release 49, 2015/02/17) |
| YHRD | Egypt | Qena | Egyptian | 52 | 34 | Yes | YP000332 (http://yhrd.org - Release 49, 2015/02/17) |
| YHRD | Egypt | Sohag | Egyptian | 52 | 40 | Yes | YP000382 (http://yhrd.org - Release 49, 2015/02/17) |
| YHRD | Estonia | Tartu | Estonian | 125 | 123 | Yes | YP000403 (http://yhrd.org - Release 49, 2015/02/17) |
| YHRD | Ethiopia | Nationwide | Amharic | 114 | 114 | No | YP000142 (http://yhrd.org - Release 49, 2015/02/17) |
| YHRD | F.Y.R.O.M (Macedonia) | Nationwide | Macedonian | 201 | 193 | Yes | YP000233 (http://yhrd.org - Release 49, 2015/02/17) |
| YHRD | Finland | Nationwide | Finnish | 269 | 269 | Yes | YP000143 (http://yhrd.org - Release 49, 2015/02/17) |
| YHRD | Finland | Turku | Finnish | 162 | 161 | Yes | YP000905 (http://yhrd.org - Release 49, 2015/02/17) |
| YHRD | France | Clermont-Ferrand | French | 89 | 84 | Yes | YP000871 (http://yhrd.org - Release 49, 2015/02/17) |
| YHRD | France | Lille | French | 70 | 70 | Yes | YP000872 (http://yhrd.org - Release 49, 2015/02/17) |
| YHRD | France | Marseille | French | 45 | 45 | Yes | YP000873 (http://yhrd.org - Release 49, 2015/02/17) |
| YHRD | France | Paris | French | 91 | 90 | Yes | YP000319 (http://yhrd.org - Release 49, 2015/02/17) |
| YHRD | France | Rennes | French | 115 | 114 | Yes | YP000874 (http://yhrd.org - Release 49, 2015/02/17) |
| YHRD | France | Strasbourg | French | 81 | 78 | Yes | YP000390 (http://yhrd.org - Release 49, 2015/02/17) |
| YHRD | France | Toulouse | French | 67 | 63 | Yes | YP000875 (http://yhrd.org - Release 49, 2015/02/17) |
| YHRD | Germany | Berlin | German | 204 | 200 | Yes | YP000037 (http://yhrd.org - Release 49, 2015/02/17) |
| YHRD | Germany | Cologne | German | 652 | 643 | Yes | YP000099 (http://yhrd.org - Release 49, 2015/02/17) |
| YHRD | Germany | Freiburg | German | 260 | 255 | Yes | YP000148 (http://yhrd.org - Release 49, 2015/02/17) |
| YHRD | Germany | Greifswald | German | 176 | 173 | Yes | YP000158 (http://yhrd.org - Release 49, 2015/02/17) |
| YHRD | Germany | Leipzig | German | 303 | 296 | Yes | YP000209 (http://yhrd.org - Release 49, 2015/02/17) |
| YHRD | Germany | Rostock | German | 601 | 598 | Yes | YP000349 (http://yhrd.org - Release 49, 2015/02/17) |
| YHRD | Germany | Stuttgart | German | 623 | 614 | Yes | YP000391 (http://yhrd.org - Release 49, 2015/02/17) |
| YHRD | Germany | Upper Bavaria | German | 200 | 196 | Yes | YP000829 (http://yhrd.org - Release 49, 2015/02/17) |
| YHRD | Greece | Athens | Greek | 148 | 142 | Yes | YP000025 (http://yhrd.org - Release 49, 2015/02/17) |
| YHRD | Greece | Nationwide | Romani | 57 | 57 | No | YP001071 (http://yhrd.org - Release 49, 2015/02/17) |
| YHRD | Greece | Nationwide | Greek | 199 | 191 | Yes | YP000695 (http://yhrd.org - Release 49, 2015/02/17) |
| YHRD | Greece | Northern Greece | Greek | 191 | 169 | Yes | YP000523 (http://yhrd.org - Release 49, 2015/02/17) |
| YHRD | Hungary | Baranya | Romani | 78 | 78 | No | YP000030 (http://yhrd.org - Release 49, 2015/02/17) |
| YHRD | Hungary | Budapest | Hungarian | 139 | 133 | Yes | YP000062 (http://yhrd.org - Release 49, 2015/02/17) |
| YHRD | Hungary | Debrecen | Romani | 44 | 39 | No | YP000114 (http://yhrd.org - Release 49, 2015/02/17) |
| YHRD | Hungary | Nationwide | Hungarian | 308 | 303 | Yes | YP000175 (http://yhrd.org - Release 49, 2015/02/17) |
| YHRD | Hungary | Nationwide | Romani | 291 | 291 | No | YP000176 (http://yhrd.org - Release 49, 2015/02/17) |
| YHRD | Iran | Ahvaz | Arab | 47 | 33 | Yes | YP000513 (http://yhrd.org - Release 49, 2015/02/17) |
| YHRD | Iran | Central Iran | Iranian | 154 | 152 | Yes | YP000821 (http://yhrd.org - Release 49, 2015/02/17) |
| YHRD | Iran | Izeh | Bakthiari | 50 | 45 | Yes | YP000512 (http://yhrd.org - Release 49, 2015/02/17) |
| YHRD | Iran | Mashhad | Iranian | 127 | 127 | Yes | YP000925 (http://yhrd.org - Release 49, 2015/02/17) |
| YHRD | Iran | Rasht | Gilaki | 47 | 42 | Yes | YP000517 (http://yhrd.org - Release 49, 2015/02/17) |
| YHRD | Iran | Sari | Mazandarani | 50 | 44 | Yes | YP000516 (http://yhrd.org - Release 49, 2015/02/17) |
| YHRD | Iran | Zahedan Sistan Baluchestan | Iranian | 102 | 102 | Yes | YP000923 (http://yhrd.org - Release 49, 2015/02/17) |
| YHRD | Iraq | Nationwide | Iraqi | 124 | 99 | Yes | YP000890 (http://yhrd.org - Release 49, 2015/02/17) |
| YHRD | Ireland | Nationwide | Irish | 226 | 225 | Yes | YP000188 (http://yhrd.org - Release 49, 2015/02/17) |
| YHRD | Israel | Northern Israel | Druze | 40 | 40 | Yes | YP000960 (http://yhrd.org - Release 49, 2015/02/17) |
| YHRD | Israel/Palestine | Israel and Palestinian Authority Area Arab | Arab | 163 | 163 | Yes | YP000694 (http://yhrd.org - Release 49, 2015/02/17) |
| YHRD | Italy | Belvedere | Italian | 30 | 30 | Yes | YP000770 (http://yhrd.org - Release 49, 2015/02/17) |
| YHRD | Italy | Bergamo | Italian | 150 | 148 | Yes | YP000980 (http://yhrd.org - Release 49, 2015/02/17) |
| YHRD | Italy | Brescia | Italian | 248 | 246 | Yes | YP000057 (http://yhrd.org - Release 49, 2015/02/17) |
| YHRD | Italy | Calabria | Arbereshe | 90 | 86 | Yes | YP000035 (http://yhrd.org - Release 49, 2015/02/17) |
| YHRD | Italy | Casentino | Italian | 50 | 47 | Yes | YP000988 (http://yhrd.org - Release 49, 2015/02/17) |
| YHRD | Italy | Nationwide | Italian | 152 | 147 | Yes | YP000981 (http://yhrd.org - Release 49, 2015/02/17) |
| YHRD | Italy | La Spezia | Italian | 45 | 43 | Yes | YP000768 (http://yhrd.org - Release 49, 2015/02/17) |
| YHRD | Italy | Latium | Italian | 78 | 71 | Yes | YP000206 (http://yhrd.org - Release 49, 2015/02/17) |
| YHRD | Italy | Lombardy | Italian | 70 | 66 | Yes | YP000218 (http://yhrd.org - Release 49, 2015/02/17) |
| YHRD | Italy | Marche | Italian | 165 | 156 | Yes | YP000248 (http://yhrd.org - Release 49, 2015/02/17) |
| YHRD | Italy | Marche | Moroccan | 60 | 48 | No | YP000489 (http://yhrd.org - Release 49, 2015/02/17) |
| YHRD | Italy | Marche | Tunisian | 61 | 36 | No | YP000488 (http://yhrd.org - Release 49, 2015/02/17) |
| YHRD | Italy | Modena | Italian | 130 | 130 | Yes | YP000136 (http://yhrd.org - Release 49, 2015/02/17) |
| YHRD | Italy | Northern Sardinia | Italian | 100 | 97 | Yes | YP000289 (http://yhrd.org - Release 49, 2015/02/17) |
| YHRD | Italy | Offida | Italian | 38 | 35 | Yes | YP000767 (http://yhrd.org - Release 49, 2015/02/17) |
| YHRD | Italy | Piedmont | Italian | 203 | 201 | Yes | YP000992 (http://yhrd.org - Release 49, 2015/02/17) |
| YHRD | Italy | Puglia | Italian | 160 | 146 | Yes | YP000330 (http://yhrd.org - Release 49, 2015/02/17) |
| YHRD | Italy | Ravenna | Italian | 382 | 364 | Yes | YP000336 (http://yhrd.org - Release 49, 2015/02/17) |
| YHRD | Italy | Reggio di Calabria | Italian | 74 | 73 | Yes | YP000543 (http://yhrd.org - Release 49, 2015/02/17) |
| YHRD | Italy | Sardinia | Italian | 319 | 299 | Yes | YP000529 (http://yhrd.org - Release 49, 2015/02/17) |
| YHRD | Italy | Sicily | Italian | 101 | 101 | Yes | YP000375 (http://yhrd.org - Release 49, 2015/02/17) |
| YHRD | Italy | Sicily | Arbereshe | 42 | 42 | No | YP001034 (http://yhrd.org - Release 49, 2015/02/17) |
| YHRD | Italy | Trapani | Italian | 39 | 33 | Yes | YP000415 (http://yhrd.org - Release 49, 2015/02/17) |
| YHRD | Italy | Tuscany | Italian | 59 | 58 | Yes | YP000424 (http://yhrd.org - Release 49, 2015/02/17) |
| YHRD | Italy | Udine | Italian | 47 | 45 | Yes | YP000765 (http://yhrd.org - Release 49, 2015/02/17) |
| YHRD | Italy | Verona | Italian | 335 | 325 | Yes | YP000441 (http://yhrd.org - Release 49, 2015/02/17) |
| YHRD | Jordan | Nationwide | Arab-Adnanit | 50 | 50 | Yes | YP000572 (http://yhrd.org - Release 49, 2015/02/17) |
| YHRD | Jordan | Nationwide | Arab-Qahtanit | 114 | 114 | Yes | YP000571 (http://yhrd.org - Release 49, 2015/02/17) |
| YHRD | Kazakhstan | East Kazahstan | Kazakh | 67 | 67 | No | YP000745 (http://yhrd.org - Release 49, 2015/02/17) |
| YHRD | Kazakhstan | South Kazakhstan | Kazakh | 99 | 99 | Yes | YP000773 (http://yhrd.org - Release 49, 2015/02/17) |
| YHRD | Kuwait | Nationwide | Kuwaiti | 249 | 145 | Yes | YP000538 (http://yhrd.org - Release 49, 2015/02/17) |
| YHRD | Kuwait | Kuwait City | Arab | 285 | 160 | Yes | YP000802 (http://yhrd.org - Release 49, 2015/02/17) |
| YHRD | Latvia | Riga | Latvian | 139 | 138 | Yes | YP000342 (http://yhrd.org - Release 49, 2015/02/17) |
| YHRD | Lebanon | Beirut | Lebanese | 555 | 421 | Yes | YP000823 (http://yhrd.org - Release 49, 2015/02/17) |
| YHRD | Libya | Banghazi | Arab | 238 | 238 | Yes | YP000737 (http://yhrd.org - Release 49, 2015/02/17) |
| YHRD | Libya | Tripoli | Libyan | 175 | 122 | Yes | YP000728 (http://yhrd.org - Release 49, 2015/02/17) |
| YHRD | Lithuania | Vilnius | Lithuanian | 84 | 84 | Yes | YP000445 (http://yhrd.org - Release 49, 2015/02/17) |
| YHRD | Lithuania | Nationwide | Lithuanian | 194 | 84 | Yes | YP000710 (http://yhrd.org - Release 49, 2015/02/17) |
| YHRD | Morocco | Figuig | Berber | 52 | 44 | No | YP000507 (http://yhrd.org - Release 49, 2015/02/17) |
| YHRD | Morocco | Rabat | Arabs | 130 | 106 | Yes | YP000503 (http://yhrd.org - Release 49, 2015/02/17) |
| YHRD | Morocco | Rabat | Berber | 69 | 61 | No | YP000504 (http://yhrd.org - Release 49, 2015/02/17) |
| YHRD | Morocco | Rabat | Sahraouis | 68 | 61 | No | YP000505 (http://yhrd.org - Release 49, 2015/02/17) |
| YHRD | Netherlands | Friesland | Dutch | 95 | 94 | Yes | YP000149 (http://yhrd.org - Release 49, 2015/02/17) |
| YHRD | Netherlands | Nationwide | Dutch | 2085 | 2039 | Yes | YP000279 (http://yhrd.org - Release 49, 2015/02/17) |
| YHRD | Netherlands | Noord-Brabant | Dutch | 122 | 120 | Yes | YP000702 (http://yhrd.org - Release 49, 2015/02/17) |
| YHRD | Norway | Bergen | Norwegian | 85 | 83 | Yes | YP000036 (http://yhrd.org - Release 49, 2015/02/17) |
| YHRD | Norway | Central Norway | Norwegian | 285 | 281 | Yes | YP000083 (http://yhrd.org - Release 49, 2015/02/17) |
| YHRD | Norway | Eastern Norway | Norwegian | 435 | 431 | Yes | YP000124 (http://yhrd.org - Release 49, 2015/02/17) |
| YHRD | Norway | Northern Norway | Norwegian | 339 | 337 | Yes | YP000287 (http://yhrd.org - Release 49, 2015/02/17) |
| YHRD | Norway | Oslo | Norwegian | 95 | 92 | Yes | YP000304 (http://yhrd.org - Release 49, 2015/02/17) |
| YHRD | Norway | Southern Norway | Norwegian | 65 | 65 | Yes | YP000387 (http://yhrd.org - Release 49, 2015/02/17) |
| YHRD | Norway | Western Norway | Norwegian | 270 | 266 | Yes | YP000457 (http://yhrd.org - Release 49, 2015/02/17) |
| YHRD | Poland | Bialystok | Polish | 150 | 150 | Yes | YP000040 (http://yhrd.org - Release 49, 2015/02/17) |
| YHRD | Poland | Gdansk | Polish | 2036 | 2020 | Yes | YP000152 (http://yhrd.org - Release 49, 2015/02/17) |
| YHRD | Poland | Krakow | Polish | 157 | 155 | Yes | YP000203 (http://yhrd.org - Release 49, 2015/02/17) |
| YHRD | Poland | Poznan | Polish | 244 | 244 | Yes | YP000840 (http://yhrd.org - Release 49, 2015/02/17) |
| YHRD | Poland | Southern Poland | Polish | 514 | 501 | Yes | YP000522 (http://yhrd.org - Release 49, 2015/02/17) |
| YHRD | Poland | Warsaw | Polish | 495 | 491 | Yes | YP000455 (http://yhrd.org - Release 49, 2015/02/17) |
| YHRD | Poland | Wroclaw | Polish | 591 | 583 | Yes | YP000459 (http://yhrd.org - Release 49, 2015/02/17) |
| YHRD | Portugal | Central Portugal | Portuguese | 386 | 372 | Yes | YP000084 (http://yhrd.org - Release 49, 2015/02/17) |
| YHRD | Portugal | Northern Portugal | Portuguese | 85 | 84 | Yes | YP000288 (http://yhrd.org - Release 49, 2015/02/17) |
| YHRD | Portugal | Southern Portugal | Portuguese | 80 | 78 | Yes | YP000388 (http://yhrd.org - Release 49, 2015/02/17) |
| YHRD | Romania | Moldavia | Romanian | 40 | 39 | Yes | YP000518 (http://yhrd.org - Release 49, 2015/02/17) |
| YHRD | Romania | Nationwide | Romanian | 135 | 135 | Yes | YP000348 (http://yhrd.org - Release 49, 2015/02/17) |
| YHRD | Romania | Nationwide | Romani | 83 | 83 | No | YP001072 (http://yhrd.org - Release 49, 2015/02/17) |
| YHRD | Romania | Wallachia | Romanian | 96 | 96 | Yes | YP000520 (http://yhrd.org - Release 49, 2015/02/17) |
| YHRD | Rusian Federation | Novgorod | Russian | 40 | 39 | Yes | YP000291 (http://yhrd.org - Release 49, 2015/02/17) |
| YHRD | Rusian Federation | Elista | Kalmykian | 99 | 60 | Yes | YP000135 (http://yhrd.org - Release 49, 2015/02/17) |
| YHRD | Russian Federation | Archangelsk | Russian | 59 | 59 | Yes | YP000012 (http://yhrd.org - Release 49, 2015/02/17) |
| YHRD | Russian Federation | Brjansk | Russian | 43 | 41 | Yes | YP000035 (http://yhrd.org - Release 49, 2015/02/17) |
| YHRD | Russian Federation | Ivanowo | Russian | 40 | 39 | Yes | YP000191 (http://yhrd.org - Release 49, 2015/02/17) |
| YHRD | Russian Federation | Lipezk | Russian | 47 | 44 | Yes | YP000216 (http://yhrd.org - Release 49, 2015/02/17) |
| YHRD | Russian Federation | Orel | Russian | 42 | 41 | Yes | YP000302 (http://yhrd.org - Release 49, 2015/02/17) |
| YHRD | Russian Federation | Pensa | Russian | 81 | 79 | Yes | YP000324 (http://yhrd.org - Release 49, 2015/02/17) |
| YHRD | Russian Federation | Rjasan | Russian | 36 | 35 | Yes | YP000347 (http://yhrd.org - Release 49, 2015/02/17) |
| YHRD | Russian Federation | Nationwide | Yakut | 34 | 34 | No | YP001135 (http://yhrd.org - Release 49, 2015/02/17) |
| YHRD | Russian Federation | Smolensk | Russian | 43 | 42 | Yes | YP000381 (http://yhrd.org - Release 49, 2015/02/17) |
| YHRD | Russian Federation | Sverdlovsk Region | Russian | 832 | 816 | Yes | YP000726 (http://yhrd.org - Release 49, 2015/02/17) |
| YHRD | Russian Federation | Tambov | Russian | 48 | 47 | Yes | YP000401 (http://yhrd.org - Release 49, 2015/02/17) |
| YHRD | Russian Federation | Tver | Russian | 43 | 42 | Yes | YP000425 (http://yhrd.org - Release 49, 2015/02/17) |
| YHRD | Russian Federation | Vologda | Russian | 60 | 60 | Yes | YP000450 (http://yhrd.org - Release 49, 2015/02/17) |
| YHRD | Russian Federation | Western Central Sakha Republic | Sakha | 133 | 133 | No | YP000547 (http://yhrd.org - Release 49, 2015/02/17) |
| YHRD | Serbia | Novi Sad | Rusyn | 200 | 197 | Yes | YP000678 (http://yhrd.org - Release 49, 2015/02/17) |
| YHRD | Serbia | Novi Sad | Slovak | 200 | 200 | Yes | YP000679 (http://yhrd.org - Release 49, 2015/02/17) |
| YHRD | Slovakia | Nationwide | Slovakian | 156 | 156 | Yes | YP000616 (http://yhrd.org - Release 49, 2015/02/17) |
| YHRD | Slovenia | Ljubljana | Slovenian | 104 | 97 | Yes | YP000217 (http://yhrd.org - Release 49, 2015/02/17) |
| YHRD | Spain | Alava | Basque | 43 | 42 | Yes | YP000720 (http://yhrd.org - Release 49, 2015/02/17) |
| YHRD | Spain | Alicante | Spanish | 216 | 216 | Yes | YP000800 (http://yhrd.org - Release 49, 2015/02/17) |
| YHRD | Spain | Alpujarra de la Sierra | Spanish | 50 | 50 | Yes | YP000530 (http://yhrd.org - Release 49, 2015/02/17) |
| YHRD | Spain | Andalusia | Spanish | 155 | 149 | Yes | YP001011 (http://yhrd.org - Release 49, 2015/02/17) |
| YHRD | Spain | Aragon | Spanish | 46 | 45 | Yes | YP001013 (http://yhrd.org - Release 49, 2015/02/17) |
| YHRD | Spain | Asturias | Spanish | 255 | 249 | Yes | YP000023 (http://yhrd.org - Release 49, 2015/02/17) |
| YHRD | Spain | Balearic Islands | Spanish | 139 | 137 | Yes | YP001014 (http://yhrd.org - Release 49, 2015/02/17) |
| YHRD | Spain | Barcelona | Spanish | 78 | 76 | Yes | YP000031 (http://yhrd.org - Release 49, 2015/02/17) |
| YHRD | Spain | Basque Country | Spanish | 98 | 90 | No | YP000763 (http://yhrd.org - Release 49, 2015/02/17) |
| YHRD | Spain | Basque Country | Basque | 302 | 300 | Yes | YP000290 (http://yhrd.org - Release 49, 2015/02/17) |
| YHRD | Spain | Biscay | Basque | 148 | 147 | Yes | YP000721 (http://yhrd.org - Release 49, 2015/02/17) |
| YHRD | Spain | Cantabria | Spanish | 98 | 91 | Yes | YP000073 (http://yhrd.org - Release 49, 2015/02/17) |
| YHRD | Spain | Catalonia | Spanish | 1415 | 1383 | Yes | YP001015 (http://yhrd.org - Release 49, 2015/02/17) |
| YHRD | Spain | Galicia | Spanish | 93 | 89 | Yes | YP000830 (http://yhrd.org - Release 49, 2015/02/17) |
| YHRD | Spain | Granada | Spanish | 180 | 171 | Yes | YP000733 (http://yhrd.org - Release 49, 2015/02/17) |
| YHRD | Spain | Huelva | Spanish | 167 | 164 | Yes | YP000732 (http://yhrd.org - Release 49, 2015/02/17) |
| YHRD | Spain | Madrid | Spanish | 126 | 121 | Yes | YP000236 (http://yhrd.org - Release 49, 2015/02/17) |
| YHRD | Spain | Nationwide | Spanish | 2366 | 2281 | No | YP001017 (http://yhrd.org - Release 49, 2015/02/17) |
| YHRD | Spain | Nationwide | Romani | 36 | 36 | No | YP001074 (http://yhrd.org - Release 49, 2015/02/17) |
| YHRD | Spain | Valencia | Spanish | 295 | 288 | Yes | YP000435 (http://yhrd.org - Release 49, 2015/02/17) |
| YHRD | Spain | Zaragoza | Spanish | 200 | 191 | Yes | YP000474 (http://yhrd.org - Release 49, 2015/02/17) |
| YHRD | Sudan | Nile 4th cataract | Bedouin | 64 | 35 | No | YP000697 (http://yhrd.org - Release 49, 2015/02/17) |
| YHRD | Sweden | Blekinge | Swedish | 41 | 41 | Yes | YP000046 (http://yhrd.org - Release 49, 2015/02/17) |
| YHRD | Sweden | Gotland | Swedish | 40 | 39 | Yes | YP000155 (http://yhrd.org - Release 49, 2015/02/17) |
| YHRD | Sweden | Östergötland/ Jönköping | Swedish | 40 | 38 | Yes | YP000438 (http://yhrd.org - Release 49, 2015/02/17) |
| YHRD | Sweden | Skaraborg | Swedish | 44 | 44 | Yes | YP000379 (http://yhrd.org - Release 49, 2015/02/17) |
| YHRD | Sweden | Uppsala | Swedish | 54 | 51 | Yes | YP000432 (http://yhrd.org - Release 49, 2015/02/17) |
| YHRD | Sweden | Värmland | Swedish | 42 | 42 | Yes | YP000452 (http://yhrd.org - Release 49, 2015/02/17) |
| YHRD | Sweden | Västerbotten | Swedish | 41 | 41 | Yes | YP000453 (http://yhrd.org - Release 49, 2015/02/17) |
| YHRD | Switzerland | Aargau | Swiss | 505 | 499 | Yes | YP000891 (http://yhrd.org - Release 49, 2015/02/17) |
| YHRD | Switzerland | Basel | Swiss | 138 | 122 | Yes | YP000911 (http://yhrd.org - Release 49, 2015/02/17) |
| YHRD | Switzerland | Lausanne | Swiss | 100 | 97 | Yes | YP000207 (http://yhrd.org - Release 49, 2015/02/17) |
| YHRD | Switzerland | Nationwide | Swiss | 150 | 147 | Yes | YP000395 (http://yhrd.org - Release 49, 2015/02/17) |
| YHRD | Tunisia | Sousse | Tunisian | 218 | 153 | Yes | YP000787 (http://yhrd.org - Release 49, 2015/02/17) |
| YHRD | Turkey | East Anatolia | Turkish | 37 | 36 | Yes | YP001086 (http://yhrd.org - Release 49, 2015/02/17) |
| YHRD | Turkey | Çukurova | Turkish | 249 | 207 | Yes | YP000717 (http://yhrd.org - Release 49, 2015/02/17) |
| YHRD | Turkey | Marmara Region | Turkish | 385 | 329 | Yes | YP000083 (http://yhrd.org - Release 49, 2015/02/17) |
| YHRD | Turkey | Southeastern Anatolia | Turkish | 150 | 125 | Yes | YP000771 (http://yhrd.org - Release 49, 2015/02/17) |
| YHRD | Turkey | Nationwide | Turkish | 320 | 296 | Yes | YP000423 (http://yhrd.org - Release 49, 2015/02/17) |
| YHRD | Turkey | Black Sea Region | Turkish | 103 | 87 | Yes | YP001084 (http://yhrd.org - Release 49, 2015/02/17) |
| YHRD | Turkey | Dogu, Central Anatolia | Kurdish | 31 | 31 | Yes | YP000525 (http://yhrd.org - Release 49, 2015/02/17) |
| YHRD | Turkey | Eskikoy, Central Anatolia | Turkish | 30 | 30 | Yes | YP000527 (http://yhrd.org - Release 49, 2015/02/17) |
| YHRD | Turkey | Gocmen, Central Anatolia | Afsar | 30 | 30 | Yes | YP000528 (http://yhrd.org - Release 49, 2015/02/17) |
| YHRD | Turkey | Merkez, Central Anatolia | Kurdish | 49 | 49 | Yes | YP000526 (http://yhrd.org - Release 49, 2015/02/17) |
| YHRD | Ukraine | Lviv | Ukrainian | 154 | 151 | Yes | YP000230 (http://yhrd.org - Release 49, 2015/02/17) |
| YHRD | Ukraine | Nationwide | Ukrainian | 40 | 40 | Yes | YP001075 (http://yhrd.org - Release 49, 2015/02/17) |
| YHRD | United Arab Emirates | Abu Dhabi | Arab | 191 | 109 | Yes | YP000803 (http://yhrd.org - Release 49, 2015/02/17) |
| YHRD | United Arab Emirates | Nationwide | Arab | 278 | 159 | Yes | YP001070 (http://yhrd.org - Release 49, 2015/02/17) |
| YHRD | United Kingdom | Central England | English | 81 | 80 | Yes | YP000907 (http://yhrd.org - Release 49, 2015/02/17) |
| YHRD | United Kingdom | Southern England | English | 114 | 113 | Yes | YP000908 (http://yhrd.org - Release 49, 2015/02/17) |
| YHRD | United Kingdom | London | English | 162 | 159 | Yes | YP000219 (http://yhrd.org - Release 49, 2015/02/17) |
| YHRD | United Kingdom | Nationwide | British | 47 | 44 | No | YP000906 (http://yhrd.org - Release 49, 2015/02/17) |
| YHRD | United Kingdom | Wales | Welsh | 118 | 117 | Yes | YP000909 (http://yhrd.org - Release 49, 2015/02/17) |
| YHRD | Yemen | Sanaa | Yemeni | 128 | 46 | Yes | YP000354 (http://yhrd.org - Release 49, 2015/02/17) |
| Comparative database | Austria | Reutte | Tyrolean | 259 | 253 | Yes | ([1](#_ENREF_1)) |
| Comparative database | Belgium | Antwerpen | Belgian | 206 | 202 | Yes | ([1](#_ENREF_1)) |
| Comparative database | Belgium | Vlaams-Brabant | Belgian | 105 | 101 | Yes | ([1](#_ENREF_1)) |
| Comparative database | Bosnia and Herzegovina | Nationwide | Bosnian | 100 | 94 | Yes | ([1](#_ENREF_1)) |
| Comparative database | Bulgaria | Nationwide | Bulgarian | 195 | 195 | Yes | ([2](#_ENREF_2)) |
| Comparative database | Croatia | Nationwide | Croatian | 125 | 119 | Yes | ([1](#_ENREF_1)) |
| Comparative database | Croatia | Zagreb | Croatian | 114 | 113 | Yes | ([3](#_ENREF_3)) |
| Comparative database | Cyprus | Nationwide | Greek Cypriots | 344 | 299 | Yes | This study |
| Comparative database | Cyprus | Nationwide | Greek Cypriots | 574 | 469 | No | ([4](#_ENREF_4)) |
| Comparative database | Cyprus | Nationwide | Turkish Cypriots | 380 | 378 | Yes | ([5](#_ENREF_5)); ([6](#_ENREF_6)) |
| Comparative database | Czech Republic | Bohemia | Czech | 72 | 70 | Yes | ([1](#_ENREF_1)) |
| Comparative database | Czech Republic | Moravia | Czech | 42 | 39 | Yes | ([1](#_ENREF_1)) |
| Comparative database | Denmark | Copenhagen | Danish | 185 | 182 | Yes | ([1](#_ENREF_1)) |
| Comparative database | Egypt | Nationwide | Egyptian | 91 | 90 | Yes | ([7](#_ENREF_7)) |
| Comparative database | Estonia | Nationwide | Estonian | 125 | 123 | Yes | ([1](#_ENREF_1)) |
| Comparative database | F.Y.R.O.M (Macedonia) | Nationwide | Macedonian | 101 | 96 | Yes | ([1](#_ENREF_1)) |
| Comparative database | Finland | Nationwide | Finnish | 254 | 254 | Yes | ([1](#_ENREF_1)) |
| Comparative database | Finland | Turku | Finnish | 162 | 161 | Yes | ([1](#_ENREF_1)) |
| Comparative database | Germany | Berlin | German | 131 | 129 | Yes | ([1](#_ENREF_1)) |
| Comparative database | Germany | Freiburg | German | 260 | 255 | Yes | ([1](#_ENREF_1)) |
| Comparative database | Germany | Leipzig | German | 303 | 296 | Yes | ([1](#_ENREF_1)) |
| Comparative database | Germany | Mecklenburg-Vorpommern | German | 176 | 173 | Yes | ([1](#_ENREF_1)) |
| Comparative database | Germany | Rostock | German | 530 | 527 | Yes | ([1](#_ENREF_1)) |
| Comparative database | Germany | Stuttgart | German | 118 | 116 | Yes | ([1](#_ENREF_1)) |
| Comparative database | Germany | Upper Bavaria | German | 200 | 196 | Yes | ([1](#_ENREF_1)) |
| Comparative database | Greece | Nationwide | Greek | 214 | 202 | Yes | ([1](#_ENREF_1)) |
| Comparative database | Greece | Northern Greece | Greek | 191 | 172 | Yes | ([8](#_ENREF_8)) |
| Comparative database | Hungary | Baranya County | Romani | 53 | 53 | No | ([1](#_ENREF_1)) |
| Comparative database | Hungary | Budapest | Hungarian | 100 | 94 | Yes | ([1](#_ENREF_1)) |
| Comparative database | Hungary | Nationwide | Hungarian | 143 | 138 | Yes | ([1](#_ENREF_1)) |
| Comparative database | Hungary | Nationwide | Romani | 101 | 101 | No | ([1](#_ENREF_1)) |
| Comparative database | Iran | Nationwide | Iranian | 160 | 151 | Yes | ([9](#_ENREF_9)) |
| Comparative database | Iraq | Nationwide | Iraqi | 124 | 99 | Yes | ([9](#_ENREF_9)) |
| Comparative database | Israel | Nationwide | Israeli Christian | 44 | 33 | No | ([10](#_ENREF_10)) |
| Comparative database | Israel | Nationwide | Israeli Muslim | 119 | 109 | No | ([10](#_ENREF_10)) |
| Comparative database | Italy | Brescia | Italian | 124 | 123 | Yes | ([1](#_ENREF_1)) |
| Comparative database | Italy | Benetutti | Sardinian | 48 | 43 | Yes | ([11](#_ENREF_11)) |
| Comparative database | Italy | Carloforte | Sardinian | 41 | 40 | Yes | ([11](#_ENREF_11)) |
| Comparative database | Italy | Central Sardinia | Sardinian | 44 | 41 | Yes | ([11](#_ENREF_11)) |
| Comparative database | Italy | Desulo | Sardinian | 50 | 45 | Yes | ([11](#_ENREF_11)) |
| Comparative database | Italy | North Sardinia | Sardinian | 37 | 36 | Yes | ([11](#_ENREF_11)) |
| Comparative database | Italy | South Sardinia | Sardinian | 48 | 46 | Yes | ([11](#_ENREF_11)) |
| Comparative database | Italy | Belvedere, Calabria | Italian | 30 | 30 | Yes | ([1](#_ENREF_1)) |
| Comparative database | Italy | Central Liguria | Italian | 46 | 44 | Yes | ([12](#_ENREF_12)) |
| Comparative database | Italy | Central Marche | Italian | 38 | 35 | Yes | ([12](#_ENREF_12)) |
| Comparative database | Italy | East Friuli Venezia-Giulia Udine | Italian | 47 | 45 | Yes | ([12](#_ENREF_12)) |
| Comparative database | Italy | Grecia Salentina South Apulia | Italian | 47 | 44 | Yes | ([12](#_ENREF_12)) |
| Comparative database | Italy | Marche | Italian | 100 | 97 | Yes | ([1](#_ENREF_1)) |
| Comparative database | Italy | Milano | Italian | 70 | 66 | Yes | ([1](#_ENREF_1)) |
| Comparative database | Italy | Modena | Italian | 130 | 49 | Yes | ([13](#_ENREF_13)) |
| Comparative database | Italy | Northeastern Italy | Italian | 335 | 325 | Yes | ([1](#_ENREF_1)) |
| Comparative database | Italy | Puglia | Italian | 160 | 145 | Yes | ([1](#_ENREF_1)) |
| Comparative database | Italy | Ravenna | Italian | 382 | 364 | Yes | ([1](#_ENREF_1)) |
| Comparative database | Italy | Tuscany | Italian | 59 | 58 | Yes | ([1](#_ENREF_1)) |
| Comparative database | Italy | Sicily | Italian | 157 | 151 | Yes | ([1](#_ENREF_1)) |
| Comparative database | Italy | Trapani | Italian | 40 | 34 | Yes | ([12](#_ENREF_12)) |
| Comparative database | Jordan | Nationwide | Jordanian | 222 | 222 | Yes | ([7](#_ENREF_7)) |
| Comparative database | Latvia | Nationwide | Latvian | 139 | 138 | Yes | ([1](#_ENREF_1)) |
| Comparative database | Lebanon | Nationwide | Lebanese | 505 | 387 | Yes | ([1](#_ENREF_1)) |
| Comparative database | Lithuania | Nationwide | Lithuanian | 84 | 84 | Yes | ([1](#_ENREF_1)) |
| Comparative database | Morocco | Casablanca | Moroccan | 166 | 146 | Yes | ([14](#_ENREF_14)) |
| Comparative database | Morocco | Nationwide | Moroccan | 266 | 227 | Yes | ([15](#_ENREF_15)) |
| Comparative database | Netherlands | Friesland | Dutch | 95 | 94 | Yes | ([1](#_ENREF_1)) |
| Comparative database | Netherlands | Nationwide | Dutch | 2085 | 2039 | Yes | ([1](#_ENREF_1)) |
| Comparative database | Poland | Bialystok | Polish | 150 | 150 | Yes | ([1](#_ENREF_1)) |
| Comparative database | Poland | Central Poland | Polish | 102 | 102 | Yes | ([1](#_ENREF_1)) |
| Comparative database | Poland | Gdansk | Polish | 170 | 169 | Yes | ([1](#_ENREF_1)) |
| Comparative database | Poland | Krakow | Polish | 134 | 130 | Yes | ([1](#_ENREF_1)) |
| Comparative database | Poland | Poznan | Polish | 150 | 150 | Yes | ([1](#_ENREF_1)) |
| Comparative database | Poland | Wroclaw | Polish | 100 | 100 | Yes | ([1](#_ENREF_1)) |
| Comparative database | Portugal | Central Portugal | Portuguese | 83 | 80 | Yes | ([1](#_ENREF_1)) |
| Comparative database | Portugal | Northeastern Portugal Sephardi | Sephardic Jews | 56 | 47 | No | ([16](#_ENREF_16)) |
| Comparative database | Portugal | Northern Portugal | Portuguese | 85 | 84 | Yes | ([1](#_ENREF_1)) |
| Comparative database | Portugal | Southern Portugal | Portuguese | 80 | 78 | Yes | ([1](#_ENREF_1)) |
| Comparative database | Romania | South-East Romania | Romanian | 122 | 122 | Yes | ([17](#_ENREF_17)) |
| Comparative database | Slovenia | Ljubljana | Slovenian | 104 | 97 | Yes | ([1](#_ENREF_1)) |
| Comparative database | Spain | Aragon | Spanish | 200 | 191 | Yes | ([1](#_ENREF_1)) |
| Comparative database | Spain | Asturias | Spanish | 256 | 241 | Yes | ([1](#_ENREF_1)) |
| Comparative database | Spain | Barcelona | Spanish | 78 | 76 | Yes | ([1](#_ENREF_1)) |
| Comparative database | Spain | Basque Country | Basque | 197 | 189 | Yes | ([18](#_ENREF_18)) |
| Comparative database | Spain | Galicia | Spanish | 46 | 44 | Yes | ([1](#_ENREF_1)) |
| Comparative database | Spain | Madrid | Spanish | 126 | 121 | Yes | ([19](#_ENREF_19)) |
| Comparative database | Sweden | Blekinge | Swedish | 41 | 41 | Yes | ([1](#_ENREF_1)) |
| Comparative database | Sweden | Gotland | Swedish | 40 | 39 | Yes | ([1](#_ENREF_1)) |
| Comparative database | Sweden | Östergötland/Jönköping | Swedish | 40 | 38 | Yes | ([1](#_ENREF_1)) |
| Comparative database | Sweden | Uppsala | Swedish | 54 | 51 | Yes | ([1](#_ENREF_1)) |
| Comparative database | Sweden | Värmland | Swedish | 42 | 42 | Yes | ([1](#_ENREF_1)) |
| Comparative database | Sweden | Västerbotten | Swedish | 41 | 41 | Yes | ([1](#_ENREF_1)) |
| Comparative database | Switzerland | Zürich | Swiss | 150 | 147 | Yes | ([1](#_ENREF_1)) |
| Comparative database | Switzerland | Basel | Swiss | 643 | 621 | Yes | ([1](#_ENREF_1)) |
| Comparative database | Switzerland | Lausanne | Swiss | 100 | 97 | Yes | ([1](#_ENREF_1)) |
| Comparative database | Turkey | Dogu, Central Anatolia | Kurdish | 31 | 31 | Yes | ([20](#_ENREF_20)) |
| Comparative database | Turkey | Eskikoy, Central Anatolia | Turkish | 30 | 30 | Yes | ([20](#_ENREF_20)) |
| Comparative database | Turkey | Gocmen, Central Anatolia | Afsar | 30 | 30 | Yes | ([20](#_ENREF_20)) |
| Comparative database | Turkey | Merkez, Central Anatolia | Kurdish | 49 | 49 | Yes | ([20](#_ENREF_20)) |
| Comparative database | Turkey | Nationwide | Turkish | 218 | 218 | Yes | ([21](#_ENREF_21)) |
| Comparative database | United Kingdom | Central England | English | 81 | 80 | Yes | ([1](#_ENREF_1)) |
| Comparative database | United Kingdom | London | English | 161 | 158 | Yes | ([1](#_ENREF_1)) |
| Comparative database | United Kingdom | South England | English | 114 | 114 | Yes | ([1](#_ENREF_1)) |
| Comparative database | United Kingdom | Nationwide | British | 47 | 44 | No | ([1](#_ENREF_1)) |
| Comparative database | United Kingdom | Nationwide | Welsh | 118 | 117 | Yes | ([1](#_ENREF_1)) |

References

1. Purps J, Siegert S, Willuweit S, Nagy M, Alves C, Salazar R, et al. A global analysis of Y-chromosomal haplotype diversity for 23 STR loci. Forensic science international Genetics. 2014;12:12-23. Epub 2014/05/24.

2. Karachanak S, Grugni V, Fornarino S, Nesheva D, Al-Zahery N, Battaglia V, et al. Y-chromosome diversity in modern Bulgarians: new clues about their ancestry. PloS one. 2013;8(3):e56779. Epub 2013/03/14.

3. Roewer L, Croucher PJ, Willuweit S, Lu TT, Kayser M, Lessig R, et al. Signature of recent historical events in the European Y-chromosomal STR haplotype distribution. Human genetics. 2005;116(4):279-91. Epub 2005/01/22.

4. Voskarides K, Mazieres S, Hadjipanagi D, Di Cristofaro J, Ignatiou A, Stefanou C, et al. Y-chromosome phylogeographic analysis of the Greek-Cypriot population reveals elements consistent with Neolithic and Bronze Age settlements. Investigative genetics. 2016;7:1. Epub 2016/02/13.

5. Terali K, Zorlu T, Bulbul O, Gurkan C. Population genetics of 17 Y-STR markers in Turkish Cypriots from Cyprus. Forensic science international Genetics. 2014;10:e1-3. Epub 2014/02/11.

6. Gurkan C, Sevay H, Demirdov DK, Soforoglu S, Ceker D, Terali K, et al. Turkish Cypriot paternal lineages bear an autochthonous character and closest resemblance to those from neighboring Near Eastern populations. Annals of human biology. 2016:1-11. Epub 2016/07/01.

7. El-Sibai M, Platt DE, Haber M, Xue Y, Youhanna SC, Wells RS, et al. Geographical structure of the Y-chromosomal genetic landscape of the Levant: a coastal-inland contrast. Annals of human genetics. 2009;73(Pt 6):568-81. Epub 2009/08/19.

8. Kovatsi L, Saunier JL, Irwin JA. Population genetics of Y-chromosome STRs in a population of Northern Greeks. Forensic science international Genetics. 2009;4(1):e21-2. Epub 2009/12/02.

9. Tabrizi AA, Hedjazi A, Kerachian MA, Honarvar Z, Dadgarmoghaddam M, Raoofian R. Genetic profile of 17 Y-chromosome STR haplotypes in East of Iran. Forensic science international Genetics. 2015;14:e6-7. Epub 2014/12/03.

10. Fernandes AT, Goncalves R, Gomes S, Filon D, Nebel A, Faerman M, et al. Y-chromosomal STRs in two populations from Israel and the Palestinian Authority Area: Christian and Muslim Arabs. Forensic science international Genetics. 2011;5(5):561-2. Epub 2010/09/17.

11. Robledo R, Mameli A, Scudiero CM, Vona G, Corrias L, Bachis V, et al. Non-random distribution of 17 Y-chromosome STR loci in different areas of Sardinia. Forensic science international Genetics. 2015;16:26-8. Epub 2014/12/17.

12. Brisighelli F, Blanco-Verea A, Boschi I, Garagnani P, Pascali VL, Carracedo A, et al. Patterns of Y-STR variation in Italy. Forensic science international Genetics. 2012;6(6):834-9. Epub 2012/04/11.

13. Ferri G, Alu M, Corradini B, Radheshi E, Beduschi G. Slow and fast evolving markers typing in Modena males (North Italy). Forensic science international Genetics. 2009;3(2):e31-3. Epub 2009/02/14.

14. Laouina A, El Houate B, Yahia H, Azeddoug H, Boulouiz R, Chbel F. Allele frequencies and population data for 17 Y-STR loci (The AmpFlSTR(R) Y-filer) in Casablanca resident population. Forensic science international Genetics. 2011;5(1):e1-3. Epub 2010/12/04.

15. Aboukhalid R, Bouabdellah M, Abbassi M, Bentayebi K, Elmzibri M, Squalli D, et al. Haplotype frequencies for 17 Y-STR loci (AmpFlSTRY-filer) in a Moroccan population sample. Forensic science international Genetics. 2010;4(3):e73-4. Epub 2010/03/11.

16. Nogueiro I, Manco L, Gomes V, Amorim A, Gusmao L. Phylogeographic analysis of paternal lineages in NE Portuguese Jewish communities. American journal of physical anthropology. 2010;141(3):373-81. Epub 2009/11/18.

17. Stanciu F, Cutar V, Pirlea S, Stoian V, Stoian IM, Sevastre O, et al. Population data for Y-chromosome haplotypes defined by 17 STRs in South-East Romania. Leg Med (Tokyo). 2010;12(5):259-64. Epub 2010/07/14.

18. Valverde L, Kohnemann S, Rosique M, Cardoso S, Zarrabeitia M, Pfeiffer H, et al. 17 Y-STR haplotype data for a population sample of Residents in the Basque Country. Forensic science international Genetics. 2012;6(4):e109-11. Epub 2012/02/22.

19. Martin P, Garcia-Hirschfeld J, Garcia O, Gusmao L, Garcia P, Albarran C, et al. A Spanish population study of 17 Y-chromosome STR loci. Forensic science international. 2004;139(2-3):231-5. Epub 2004/03/26.

20. Alakoc YD, Gokcumen O, Tug A, Gultekin T, Gulec E, Schurr TG. Y-chromosome and autosomal STR diversity in four proximate settlements in Central Anatolia. Forensic science international Genetics. 2010;4(5):e135-7. Epub 2010/05/12.

21. FiloğlunG AH, Bülbül O, Mertoğlu E, Zorlu T, Rayimoğlu G, Salih C. Polymorphism of 17 Y-chromosome STR markers in Turkiye. J For Med. 2016;30(3):213-21.
